# Supplementary figures and images for: Land use and land cover change in a tropical mountain landscape of northern Ecuador: Altitudinal patterns and driving forces
Source: PLoS One. 2022 Jul 27;17(7):e0260191. doi: 10.1371/journal.pone.0260191 (PMC9330684; doi:10.1371/journal.pone.0260191)

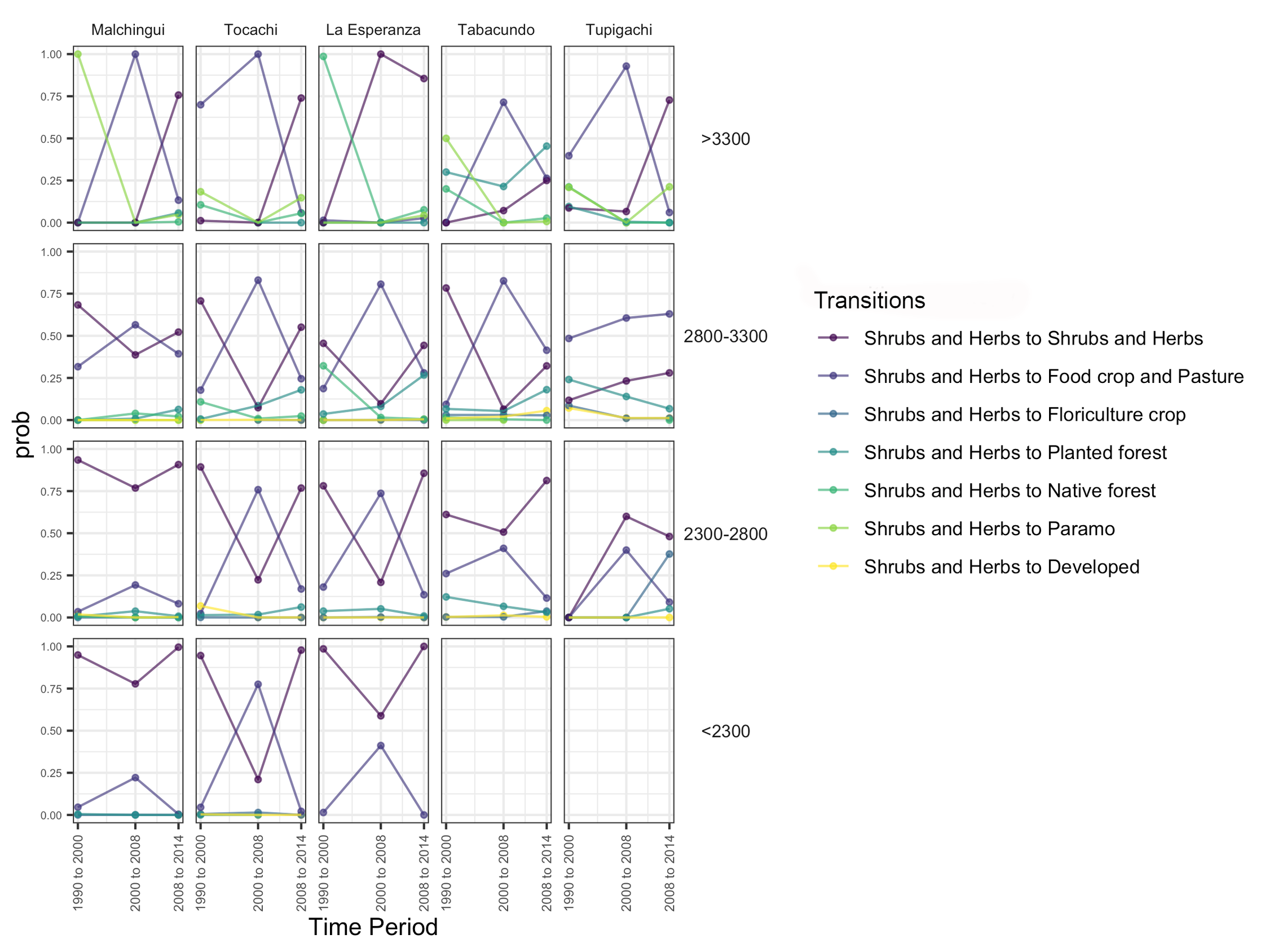

Supplement: S1 Fig — (TIF) [file pone.0260191.s001.tif]

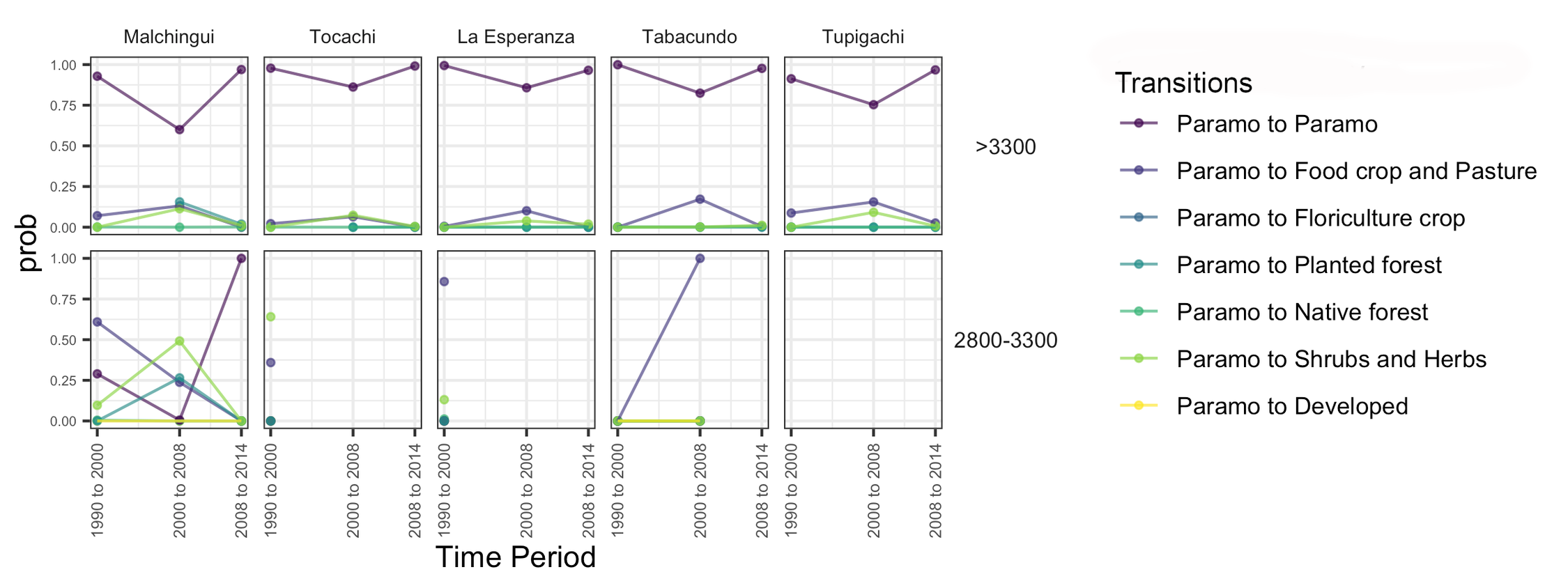

Supplement: S2 Fig — (TIF) [file pone.0260191.s002.tif]

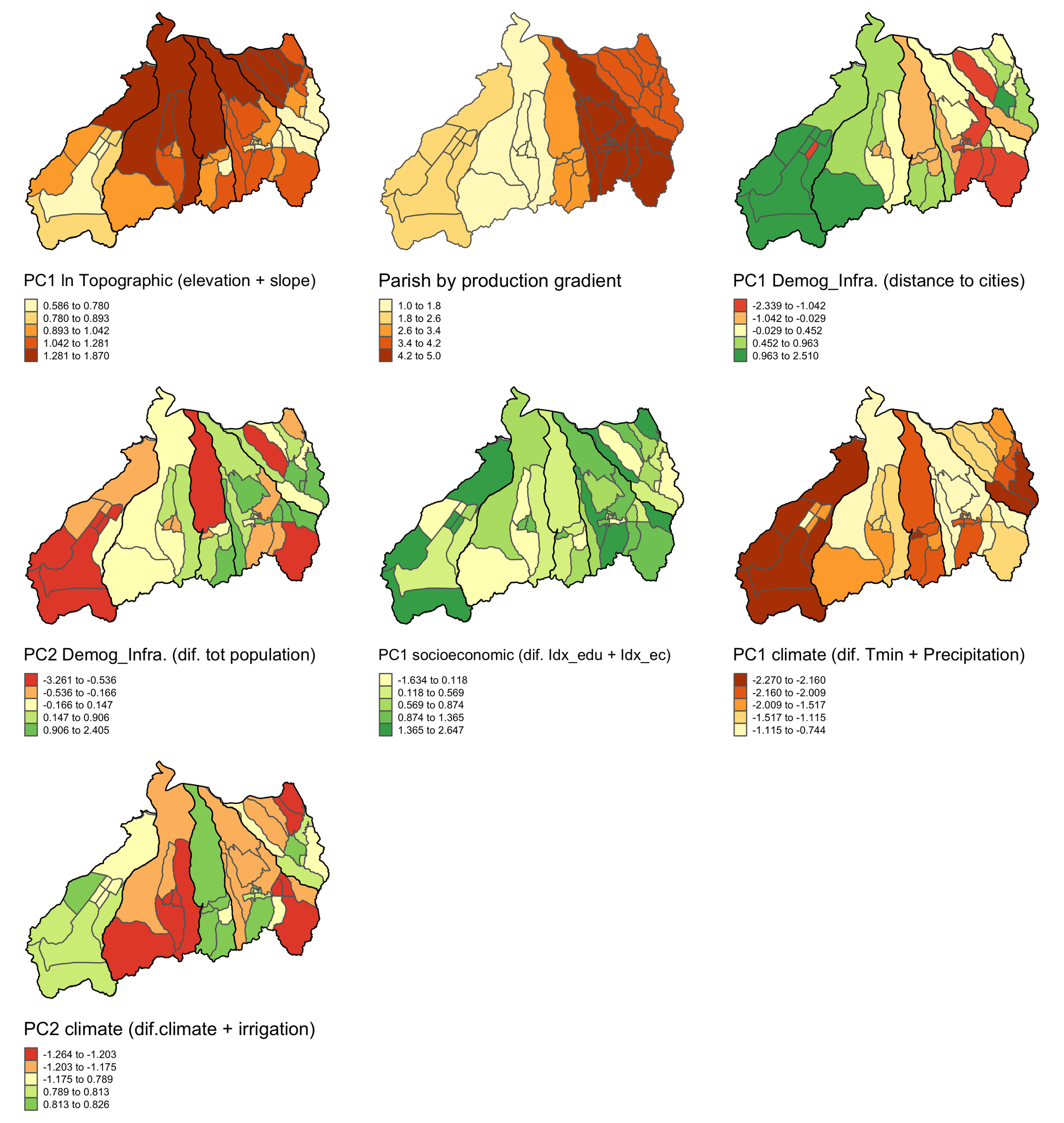

Supplement: S3 Fig — Each map represents the PC1 from the Principal Component Analysis carried out for each driver of change grouping from period 1 (1990 and 2000). (TIF) [file pone.0260191.s003.tif]

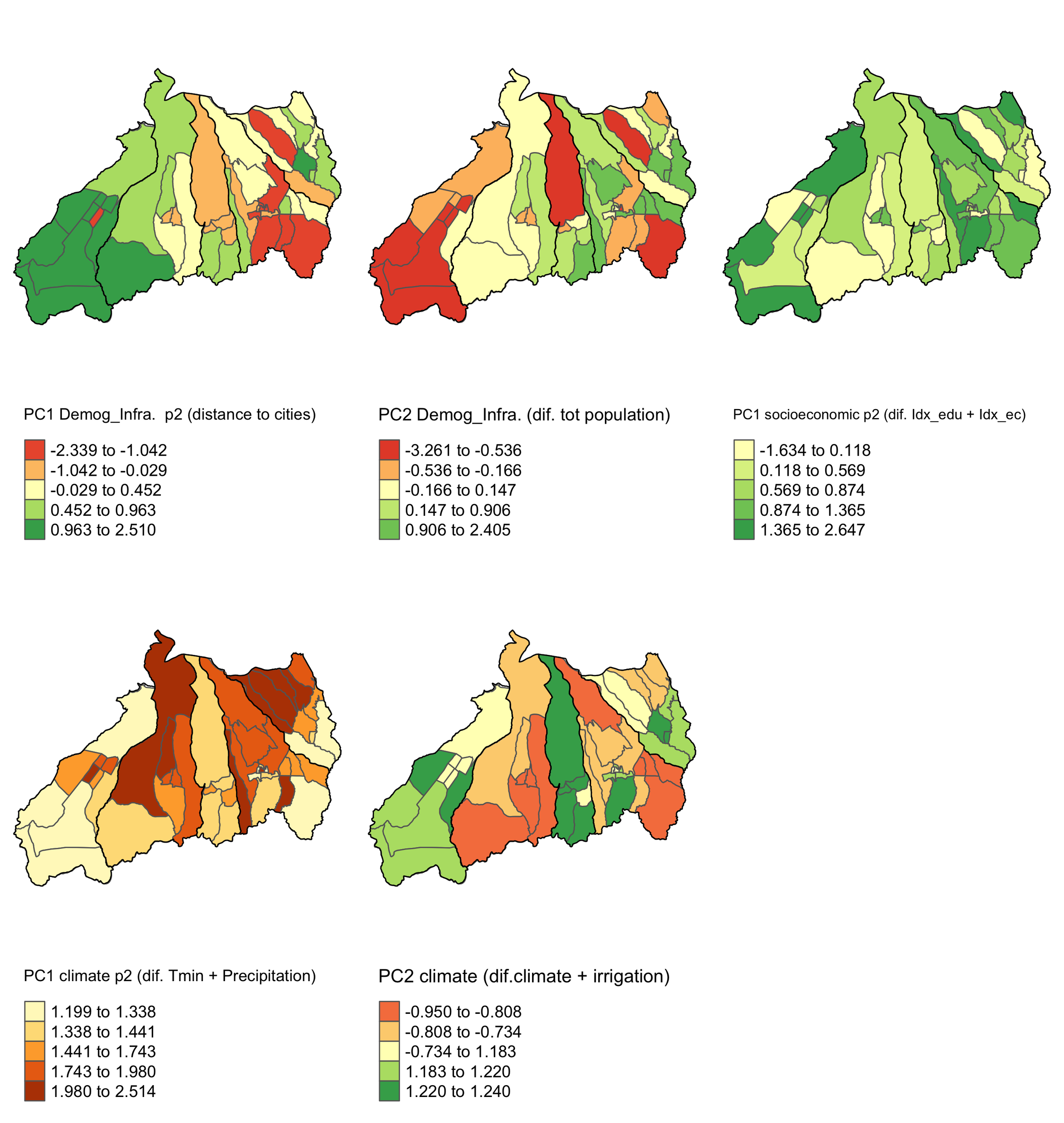

Supplement: S4 Fig — Each map represents the PC1 from the Principal Component Analysis carried out for each driver of change grouping from period 2 (2000 and 1990). (TIF) [file pone.0260191.s004.tif]

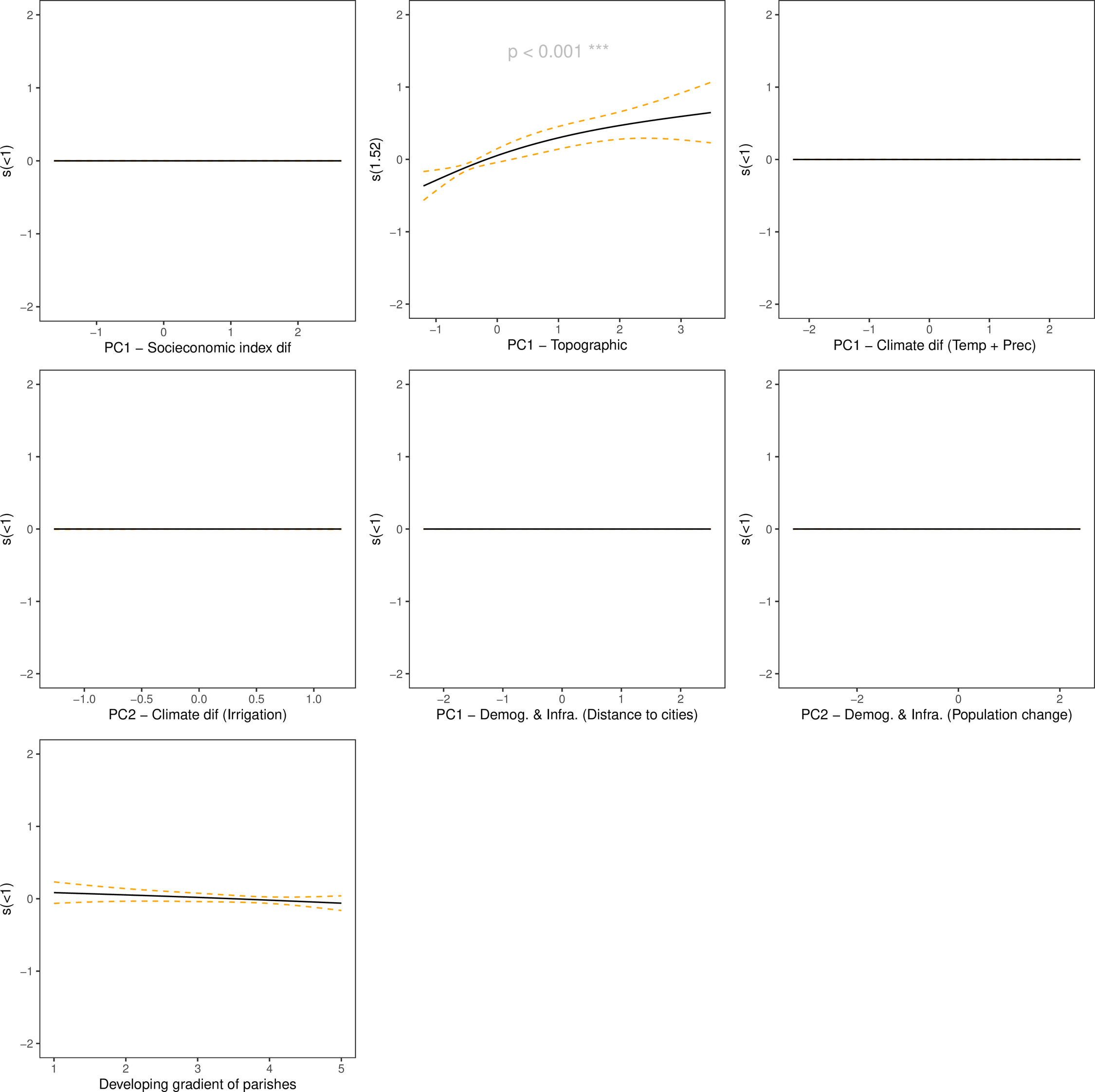

Supplement: S5 Fig — Each plot shows a covariate and their partial dependence on probability of páramo loss in the context of the model. The y axis shows the mean of the probability of native forest loss and the x axis the covariate interval. The gray area represents the 95% confidence interval. (TIF) [file pone.0260191.s005.tif]

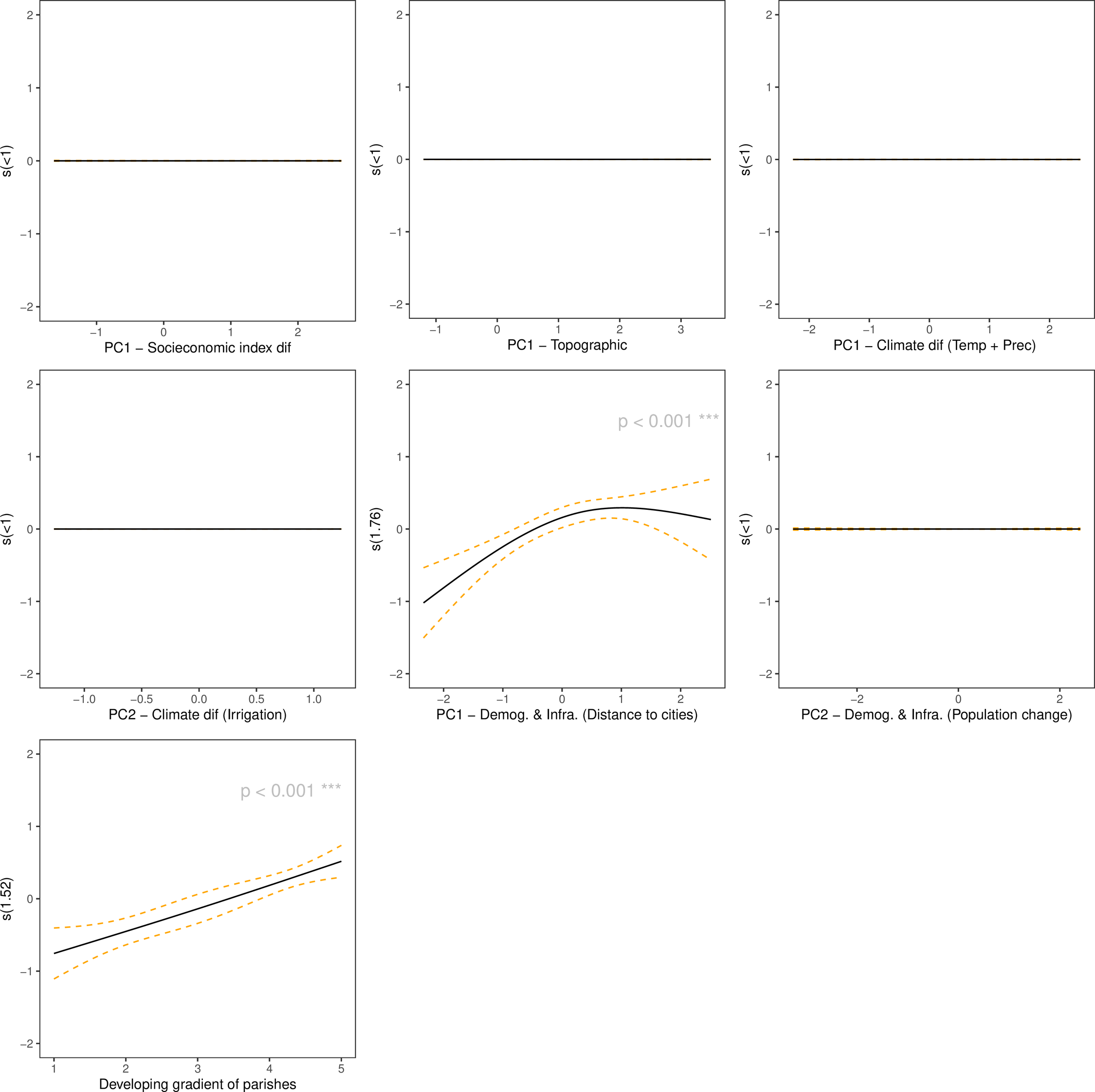

Supplement: S6 Fig — Each plot shows a covariate and their partial dependence on probability of shrubland loss in the context of the model. The y axis shows the mean of the probability of shrubland loss and the x axis the covariate interval. The gray area represents the 95% confidence interval. (TIF) [file pone.0260191.s006.tif]

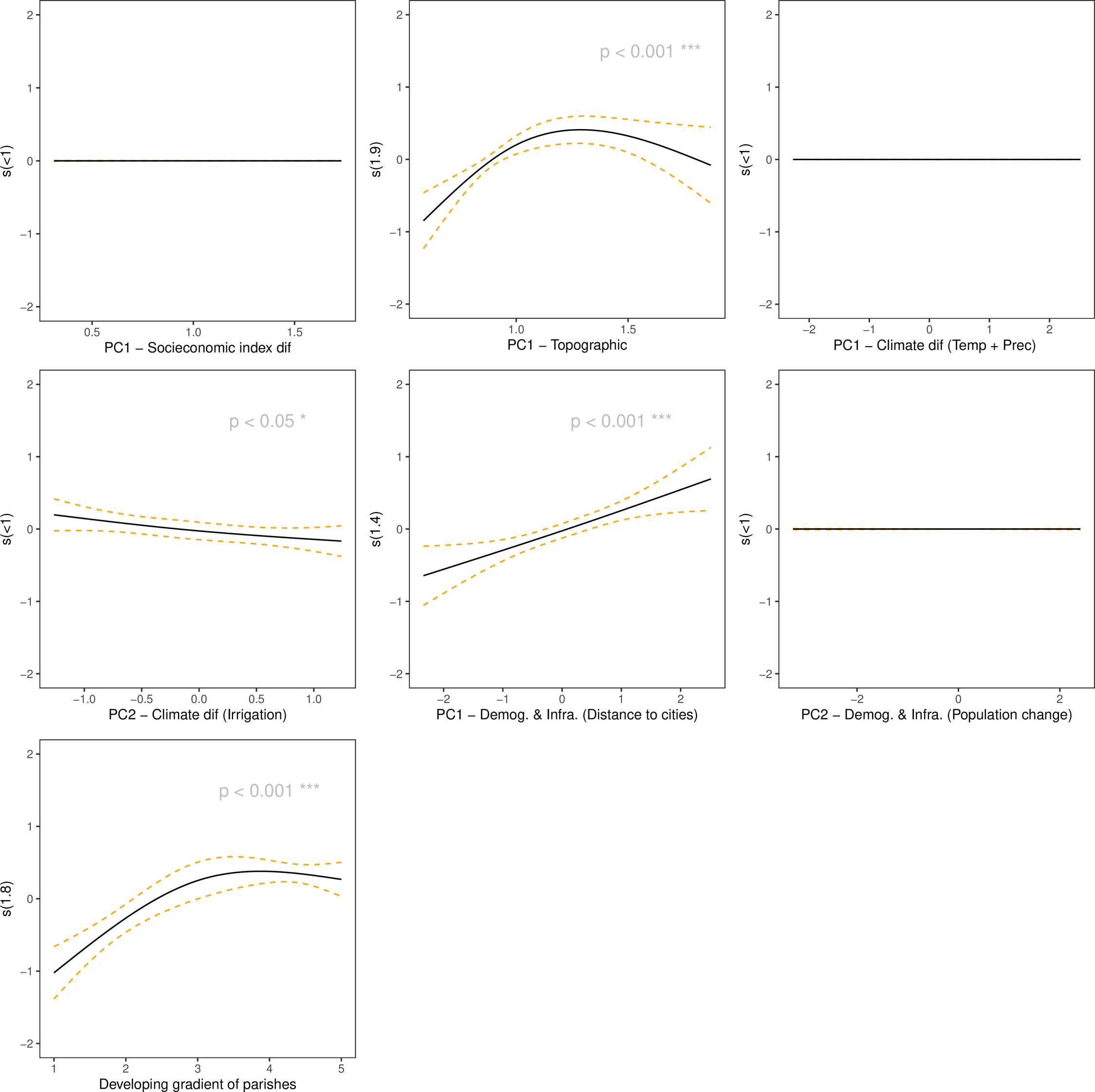

Supplement: S7 Fig — Each plot shows a covariate and their partial dependence on probability of agricultural expansion in the context of the model. The y axis shows the mean of the probability of agricultural expansion and the x axis the covariate interval. The gray area represents the 95% confidence interval. (TIF) [file pone.0260191.s007.tif]

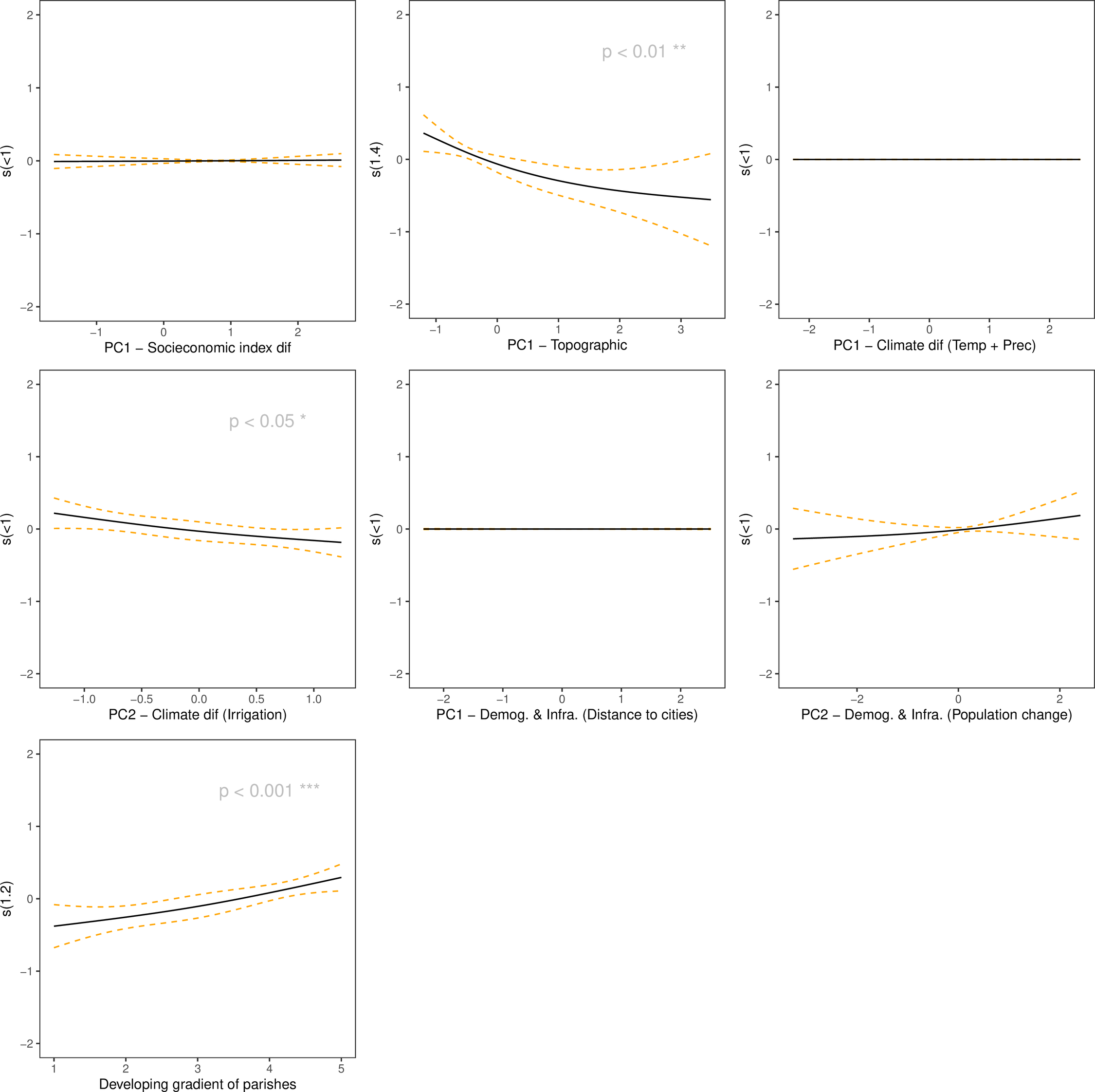

Supplement: S8 Fig — Each plot shows a covariate and their partial dependence on probability of floriculture transition in the context of the model. The y axis shows the mean of the probability of floriculture transition and the x axis the covariate interval. The gray area represents the 95% confidence interval. (TIF) [file pone.0260191.s008.tif]

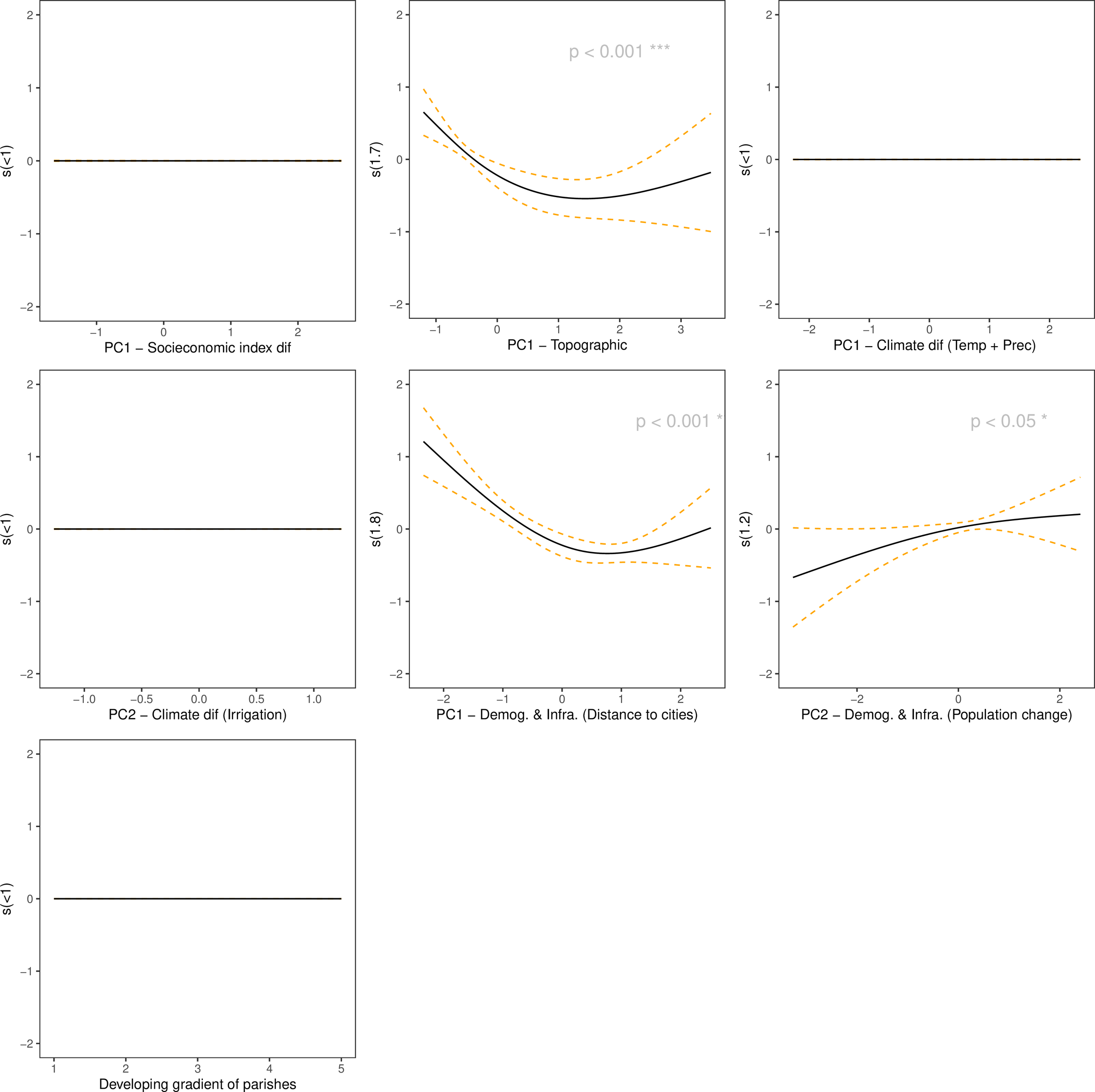

Supplement: S9 Fig — Each plot shows a covariate and their partial dependence on probability of urban transition in the context of the model. The y axis shows the mean of the probability of native forest loss and the x axis the covariate interval. The gray area represents the 95% confidence interval. (TIF) [file pone.0260191.s009.tif]
