# Supplementary material for: Land use and land cover change in a tropical mountain landscape of northern Ecuador: Altitudinal patterns and driving forces
Source: PLoS One. 2022 Jul 27;17(7):e0260191. doi: 10.1371/journal.pone.0260191 (PMC9330684; doi:10.1371/journal.pone.0260191)
Supplement: S1 Table — (PDF) [file pone.0260191.s010.pdf]

N	Class	Description
1	Developed	Land covered by concrete, including road networks, residential, industrial and commercial buildings and other infrastructures
2	Floriculture crop	Areas characterized by green house infrastructures dedicated to grow flowers
3	Agricultural land	Area under agricultural cultivation and planted pastures, or that are within a rotation cycle between them.
4	Planted forest	Anthropically established tree plantations mainly with exotic species
5	Shrubs and Herbs	Areas with a substantial component of non-tree native woody and herbaceous species, with spontaneous growth
6	Native forest	Tree ecosystem, characterized by the presence of trees of different native species, varied ages and sizes, with one or more strata.
7	Paramo	High Andean tropical vegetation characterized by dominant non-tree species that include fragments of native forest typical of the area.
8	Water bodies	Surface and associated volume of static or moving water.
