# Supplementary material for: Land use and land cover change in a tropical mountain landscape of northern Ecuador: Altitudinal patterns and driving forces
Source: PLoS One. 2022 Jul 27;17(7):e0260191. doi: 10.1371/journal.pone.0260191 (PMC9330684; doi:10.1371/journal.pone.0260191)
Supplement: Attachment — Submitted filename: Comments PONE.docx [file pone.0260191.s011.docx]

Dear Editor

I have reviewed the manuscript number PONE-D-21-35047 entitled ‘Land use land cover dynamics through time and their proximate drivers of change in a tropical mountain system: a case study in a highland landscape of northern Ecuador’. The manuscript addresses an important theme, however; the work lacks novelty and at multiple stages does not follow all the methodological steps that are crucial to maintaining the accuracy of the results. The manuscript has some major flaws in terms of data selection, accuracy and relevance of the selected data to the present-day scenario. In addition, I feel that the manuscript suffers from many issues that would require to be addressed. Theoretical development, quality of design and methods of the study are marginal. Quality of discussion is excellent whereas legitimacy of conclusions is marginal. The clarity and readability of the manuscript is good. The central argument is missing? Suggested corrections need to be incorporated before the publication of the manuscript to maintain the standard of the journal. The manuscript also needs careful proofreading to avoid grammatical errors and inconsistency as well as the latest citations and references should be included in the text. Please check the comments to the author section for detailed pointwise suggestions to improve the manuscript. I regret to inform you that the paper is unsuitable for publication in PLOS ONE. I have to give a rejection because of the insufficient data and methods used in the study.

Comments:

Dear Author/s

#### Congratulations on your good work. The manuscript PONE-D-21-35047 entitled ‘Land use land cover dynamics through time and their proximate drivers of change in a tropical mountain system: a case study in a highland landscape of northern Ecuador’ is well written. On the other hand, there are some essential comments author/s should take into consideration:

1. **Theoretical Framework:** No explicit theoretical framework and important theoretical assumptions is observed?
2. **Concepts:** A central concept of the study is not adequately and clearly defined? No new concepts have been added to the discipline.
3. **Argument:** Central argument is missing? It must be tightly and well written with examples referring from global, regional and local levels. Lots of studies have been published in recent years on a similar theme.
4. **Literature review** and use of references are inadequate. Most of the references are older than 10 years and only a few latest references are cited. Some recently updated references need to be added. Following latest references may also be cited in the manuscript for value addition:

- Mishra, P.K. Rai, A. and Rai, S.C. (2020) Land Use and Land Cover Change Detection using Geospatial Techniques in Sikkim Himalaya, India. ***Egyptian Journal of Remote Sensing and Space Sciences.*** 23 (2):133-143. doi.org/10.1016/j.ejrs.2019.02.001 IF: 5.18 (2020).
- Mishra, P.K. and Rai, A. (2020) Role of Unmanned Aerial Systems for Natural Resource Management. ***Journal of the Indian Society of Remote Sensing***. ISSN 0974-3006. DOI: 10.1007/s12524-020-01230-4 (SCOPUS). IF: 1.56 (2020).

5. The title is vague and too lengthy.
6. This study does not provide any new contribution in the field. The study lacks novelty and continuity in the analysis and provides general findings.
7. Authors have selected “Official Land Use Land Cover (LULC) maps from the Ministry of Environment of Ecuador (MAE) of four periods of time: 1990, 2000, 2008 and 2014”. However, the months and percentage of cloud cover available in satellite data are not mentioned.
8. While conducting LULC analysis, accuracy assessments play a major role in determining the overall accuracy of results. In the present manuscript accuracy assessments of maps taken from the Ministry of Environment of Ecuador is not mentioned.
9. Accuracy assessment of 5 additional topologies produced by authors is also not available in the manuscript.
10. Authors have mentioned in lines 124-125 that they digitized these 5 topologies that include planted forests, developed areas (populated zones), horticulture (areas represented by greenhouses) and natural water bodies. Line 138 authors stated that “because the study area corresponds to the major centre of floriculture production for the export market in 138 Ecuador [37,38], we added floriculture crop, as a separate typology from the agricultural land”. Areas represented by greenhouses can also have vegetations and other crops in them how was the area under floriculture estimated?
11. Digitization requires expertise in image interpretation and classes such as floriculture or horticulture are hard to map on Landsat TM data. And using Greenhouses as an indicator of horticulture and mapping entire areas of greenhouses as horticulture or floriculture without surveying is not recommended as many of them might not be in use. Results of LULC show that there is a huge growth in floriculture in the study area. However, digitizing greenhouse as floriculture is not a reliable or accurate method of mapping.
12. No Field data/assessment is available for produced LULC maps.
13. Four periods of time: 1990, 2000, 2008 and 2014 have been selected in the manuscript. From 2014 to 2021 the world has seen exponential growth in terms of LULC changes. Why not produce LULC maps of 2020 or 2021 and then do the analysis.
14. If the selected data is almost 7 years old, how do the authors think their results related to LULC change till 2014 and drivers of change are relevant today?
15. The whole manuscript is consisting of multiple grammatical and typographical errors.
16. Data sets chosen for the study has a different spatial resolution for both data. Landsat 5 (30m) and LISS 3 (23.5m). Rescaling of the data was not mentioned in the manuscript. Not rescaling both datasets on the same resolution causes inaccuracy in change detection due to different pixel sizes and even if it’s done the gap of resolution between two original data sets should be minimum. Here rescaling data from 30 m to 23.5m or vice versa would cause serious errors/loss of data.
17. Limitations of the study are not mentioned anywhere e.g., areas and reasons for misclassification.
18. References have to be rechecked as they lack similarity in writing style. In a few references, the page number is written confusingly.
19. The practical implacability of the study in any other field of work is missing. Recommendations for Future Work may add value to the manuscript.
20. The authors must check the grammar, consistency and flow of the texts in the manuscript before submitting for publication.

Best Wishes!

Dr. Prabuddh K Mishra
