# Supplementary material for: Land use and land cover change in a tropical mountain landscape of northern Ecuador: Altitudinal patterns and driving forces
Source: PLoS One. 2022 Jul 27;17(7):e0260191. doi: 10.1371/journal.pone.0260191 (PMC9330684; doi:10.1371/journal.pone.0260191)
Supplement: Attachment — Submitted filename: Response to Reviewers.docx [file pone.0260191.s012.docx]

February 22, 2022

Dear Dr. Manoj Kumar
Academic Editor
PLOS ONE

Thank you for the opportunity to submit a revised draft of the manuscript titled “Land use land cover dynamics through time and their proximate drivers of change in a tropical mountain system: a case study in a highland landscape of northern Ecuador” to PLOS ONE.

We appreciate the time and effort that you and the reviewers have dedicated to providing your valuable feedback on our manuscript. We are grateful to the reviewers for their insightful comments on our paper. We have been able to incorporate changes to reflect most of the suggestions provided by the reviewers, which have been highlighted within the manuscript.

Here is a point-by-point response to your suggestions and to the reviewers’ comments and concerns.

**Responses to the Academic Editor’s suggestions:**

*1.Please ensure that your manuscript meets PLOS ONE's style requirements, including those for file naming. The PLOS ONE style templates can be found at*
[*https://journals.plos.org/plosone/s/file?id=wjVg/PLOSOne_formatting_sample_main_body.pdf*](https://journals.plos.org/plosone/s/file?id=wjVg/PLOSOne_formatting_sample_main_body.pdf) *and*
[*https://journals.plos.org/plosone/s/file?id=ba62/PLOSOne_formatting_sample_title_authors_affiliations.pdf*](https://journals.plos.org/plosone/s/file?id=ba62/PLOSOne_formatting_sample_title_authors_affiliations.pdf)*.*

Completed. Accordingly with your suggestion, we have carefully checked the PLOS ONE style templates and we have made adjustments throughout the manuscript to fulfill the requirements.

*2.We note that you have stated that you will provide repository information for your data at acceptance. Should your manuscript be accepted for publication, we will hold it until you provide the relevant accession numbers or DOIs necessary to access your data. If you wish to make changes to your Data Availability statement, please describe these changes in your cover letter and we will update your Data Availability statement to reflect the information you provide.*

Completed. We have created an account and uploaded our datasets to Zenodo. This is one of the open access repositories recommended by PLOS ONE. We are providing below the DOI necessary to access our data:

10.5281/zenodo.5911876

*3. We note that Figures 1, 2, S3 and S4 in your submission contain [map/satellite] images which may be copyrighted. All PLOS content is published under the Creative Commons Attribution License (CC BY 4.0), which means that the manuscript, images, and Supporting Information files will be freely available online, and any third party is permitted to access, download, copy, distribute, and use these materials in any way, even commercially, with proper attribution. For these reasons, we cannot publish previously copyrighted maps or satellite images created using proprietary data, such as Google software (Google Maps, Street View, and Earth). For more information, see our copyright guidelines:* [*http://journals.plos.org/plosone/s/licenses-and-copyright*](http://journals.plos.org/plosone/s/licenses-and-copyright)*.

We require you to either (1) present written permission from the copyright holder to publish these figures specifically under the CC BY 4.0 license, or (2) remove the figures from your submission:

a) You may seek permission from the original copyright holder of Figures 1, 2, S3 and S4 to publish the content specifically under the CC BY 4.0 license.
We recommend that you contact the original copyright holder with the Content Permission Form (*[*http://journals.plos.org/plosone/s/file?id=7c09/content-permission-form.pdf*](http://journals.plos.org/plosone/s/file?id=7c09/content-permission-form.pdf)*) and the following text:
“I request permission for the open-access journal PLOS ONE to publish XXX under the Creative Commons Attribution License (CCAL) CC BY 4.0 (*[*http://creativecommons.org/licenses/by/4.0/*](http://creativecommons.org/licenses/by/4.0/)*). Please be aware that this license allows unrestricted use and distribution, even commercially, by third parties. Please reply and provide explicit written permission to publish XXX under a CC BY license and complete the attached form.”
Please upload the completed Content Permission Form or other proof of granted permissions as an "Other" file with your submission.
In the figure caption of the copyrighted figure, please include the following text: “Reprinted from [ref] under a CC BY license, with permission from [name of publisher], original copyright [original copyright year].”

b) If you are unable to obtain permission from the original copyright holder to publish these figures under the CC BY 4.0 license or if the copyright holder’s requirements are incompatible with the CC BY 4.0 license, please either i) remove the figure or ii) supply a replacement figure that complies with the CC BY 4.0 license. Please check copyright information on all replacement figures and update the figure caption with source information. If applicable, please specify in the figure caption text when a figure is similar but not identical to the original image and is therefore for illustrative purposes only.
The following resources for replacing copyrighted map figures may be helpful:

USGS National Map Viewer (public domain):* [*http://viewer.nationalmap.gov/viewer/*](http://viewer.nationalmap.gov/viewer/) *The Gateway to Astronaut Photography of Earth (public domain):* [*http://eol.jsc.nasa.gov/sseop/clickmap/*](http://eol.jsc.nasa.gov/sseop/clickmap/) *Maps at the CIA (public domain):* [*https://www.cia.gov/library/publications/the-world-factbook/index.html*](https://www.cia.gov/library/publications/the-world-factbook/index.html) *and* [*https://www.cia.gov/library/publications/cia-maps-publications/index.html*](https://www.cia.gov/library/publications/cia-maps-publications/index.html) *NASA Earth Observatory (public domain):* [*http://earthobservatory.nasa.gov/*](http://earthobservatory.nasa.gov/) *Landsat:* [*http://landsat.visibleearth.nasa.gov/*](http://landsat.visibleearth.nasa.gov/) *USGS EROS (Earth Resources Observatory and Science (EROS) Center) (public domain):* [*http://eros.usgs.gov/#*](http://eros.usgs.gov/) *Natural Earth (public domain):* [*http://www.naturalearthdata.com/*](http://www.naturalearthdata.com/)

Completed. In our revised manuscript we added another Figure (Fig 1) to depict a Conceptual framework, then, the previous Figures 1 and 2, now correspond to Figures 2 and 3. These two Figures represent maps of: the study area (new Fig 2) and the Land Use Land Cover changes through time (new Fig 3). As you suggested, we properly referred the data sources of each map in the Figure’s captions and within the map’s labels.

In addition, we searched in the journal’s repository for other articles that used georeferenced data from the official sources from Ecuador to portray maps, and we found this recent publication: Ortega-Andrade HM, Rodes Blanco M, Cisneros-Heredia DF, Guerra Arévalo N, López de Vargas-Machuca KG, Sánchez-Nivicela JC, et al. (2021) Red List assessment of amphibian species of Ecuador: A multidimensional approach for their conservation. PLoS ONE 16(5): e0251027. https://doi.org/10.1371/journal.pone.0251027. Then, we used this article as an example to follow, regarding the way the similar data sources were referred and cited.

We have also sent official communications to the Ecuadorian Governmental Institutions where geospatial data was obtained from (Ministerio del Ambiente, Agua y Transición Ecológica, y al Instituto Geográfico Militar) to ask information regarding the freely available data that we downloaded and their licenses, but we have not had had a response yet. Therefore, we cite the data sources directly, as Ortega-Andrade HM, Rodes Blanco M, Cisneros-Heredia DF, Guerra Arévalo N, López de Vargas-Machuca KG, Sánchez-Nivicela JC, et al. (2021).

We included both in the Methods and within the map’s labels of each Figure the public available URL of the web sites of the Ecuadorian Governmental Institutions where data was download for depicting the maps and running our analysis.

Two photos included in the new Figure 6 were obtained from Google Maps, and now they were replaced by three other digital photos (one depicting the developed area and two representing the floriculture crops), whose copyright holder has granted her permission to publish these figures under the CC BY 4.0 license. We uploaded the completed Content Permission Form as an "Other" file with our submission. Additionally, regarding the rest of the photos included in Figure 6 and all photos included in the new Figure 4, all were taken by the main author of this manuscript. So, we consider a written permission is not required (but if it is needed, she is willing to sign and submit the document).

Finally, Figures S3 and S4 portray results of the Principal Component Analysis of the drivers of change conducted in this research. These results were merged to the shapefiles of the census areas and spatially represented the results of our study. Therefore, they are not copyrighted figures. And the sources of information are properly cited in the Figure’s caption and elsewhere in the text.

**Responses to the reviewer’s general observation and questions**

*1. Is the manuscript technically sound, and do the data support the conclusions?
The manuscript must describe a technically sound piece of scientific research with data that supports the conclusions. Experiments must have been conducted rigorously, with appropriate controls, replication, and sample sizes. The conclusions must be drawn appropriately based on the data presented.
Reviewer #1: No
Reviewer #2: No
Reviewer #3: Yes
Reviewer #4: Partly*

We have improved the theoretical and the methodological framework of the manuscript to better connect all the elements of our study. Our previous draft did not include the Conclusion section explicitly, therefore, our revised version includes a detailed section with the main conclusions drawn from our analysis.

*2. Has the statistical analysis been performed appropriately and rigorously?
Reviewer #1: No
Reviewer #2: No
Reviewer #3: Yes
Reviewer #4: Yes*

As mentioned in our previous response, we consider that with a better description of the conceptual and methodological framework presented in the revised manuscript, the further statistical analyses are well supported and are presented in a more consistent and clearer manner. Following this scheme, the results and conclusions easily follow our framework.

*3. Have the authors made all data underlying the findings in their manuscript fully available?
The [* [*http://www.plosone.org/static/policies.action#sharing*](http://www.plosone.org/static/policies.action#sharing) *| PLOS Data policy ] requires authors to make all data underlying the findings described in their manuscript fully available without restriction, with rare exception (please refer to the Data Availability Statement in the manuscript PDF file). The data should be provided as part of the manuscript or its supporting information, or deposited to a public repository. For example, in addition to summary statistics, the data points behind means, medians and variance measures should be available. If there are restrictions on publicly sharing data—e.g. participant privacy or use of* data from a third party—those must be specified.

*Reviewer #1: No
Reviewer #2: Yes
Reviewer #3: Yes
Reviewer #4: Yes*

As mentioned in our response to the second comment from the General Editor, we have made all our data fully available in the Zenodo’s open access repository. This is the DOI 10.5281/zenodo.5911876 to access our datasets.

*4. Is the manuscript presented in an intelligible fashion and written in standard English?
PLOS ONE does not copyedit accepted manuscripts, so the language in submitted articles must be clear, correct, and unambiguous. Any typographical or grammatical errors should be corrected at revision, so please note any specific errors here.
Reviewer #1: No
Reviewer #2: Yes
Reviewer #3: Yes
Reviewer #4: No*

The manuscript was revised by English native speakers from an editing and proofreading service. Then, the English grammar of the updated version has been greatly improved.

**Responses to Specific comments from different reviewers:**

**Specific comments from Reviewer #1:**

**A. Analysis, change detection, classification methods, and plant species detection using Landsat images are not mentioned.**
What method is used for primary categorization?

*Completed.* We have added in the methods a more detailed explanation of the classification process used by the team of geographers from the Ministry of Environment and the Ministry of Agriculture, Livestock, Aquaculture and Fisheries of Ecuador to produce the LULC maps used in this article. Briefly, to generate the LULC coverages they used a supervised classification method with training data of regions of interest (ROis), using the maximum likelihood clustering algorithm. The supervised classification was performed in the ENVI software with automated image classification tools that allow the use of previously defined and refined areas or regions ROIs.

More details on the processing and classification methods used by MAE and MAGAP can be found here:

*MAE, MAGAP. Protocolo metodológico para la elaboración del mapa de cobertura y uso de la tierra del Ecuador Continental. 2015.*

*MAE. Análisis de la deforestación en el Ecuador Continental 1990 - 2014. [Internet]. Quito - Ecuador; 2016. Available from: http://suiadoc.ambiente.gob.ec/documents/10179/1149768/AnalisisDeforestacionEcuador1990_2014.pdf/8285da57-c6ca-4e82-9be7-ccea3c9317cb*

What are the indices of validation and verifications of the categorization?

To answer the reviewer’s inquiry, an expanded explanation was added in the Methods section.

Briefly, the accuracy assessment analysis of the official LULC maps encompassed an independent interpretation process, where an experienced image expert classified the coverage for each sample obtained from a stratified random sampling protocol, according to the methods proposed in the FRA global remote sensing survey (Forestry Department, 2009); this was done based on the Level 1 legend of the LULC maps of Continental Ecuador. A confusion matrix was created using the JRC Validation Tool program for the sampling areas (Simonetti, Beuchle, & Eva, 2011), and the following overall accuracy values were obtained: 69%, 73%, 76% and 85% for the years 1990, 2000, 2008 and 2014, respectively (MAE, 2016).

More details on the verification methods used by MAE and MAGAP can be found here:

Forestry Department. (2009). The 2010 global forest resources assessment remote sensing survey: an outline of the objectives, data, methods and approach. *Forest Resources Assessment Working Paper*.

Simonetti, D., Beuchle, R., & Eva, H. (2011). *User Manual for the JRC Land Cover/Use Change Validation Tool*. Retrieved from <https://publications.jrc.ec.europa.eu/repository/handle/JRC62603>

MAE. (2016). *Análisis de la deforestación en el Ecuador Continental 1990 - 2014.* Retrieved from http://suiadoc.ambiente.gob.ec/documents/10179/1149768/AnalisisDeforestacionEcuador1990_2014.pdf/8285da57-c6ca-4e82-9be7-ccea3c9317cb

*Why is the modified categorization used and for what parameters (be specific)?*

We clarified and expanded the Method’s section, explaining the reviewer’s question.

In essence, to refine the results and obtain a more accurate map of the study area, a thorough editing process of the official vector maps from the different study periods was carried out. To support this editing process, as carried out by (Madrigal-Martínez & Miralles i García, 2019), distinct secondary sources of information were revised such as field points, Google Earth images, orthophotographs and other official such as the ecosystem coverage (<http://ide.ambiente.gob.ec/mapainteractivo/>), the floriculture cadastral and other maps from the Ministry of Agriculture (<http://geoportal.agricultura.gob.ec/>). In addition, composite LANDSAT images from our study area, using radiometric enhancements and spectral band combinations were also used (Gorelick et al., 2017). From the editing process, mainly five typologies were improved. These included: planted forests, developed areas (populated zones), floriculture (areas represented by greenhouses) and natural water bodies. Following the methods proposed by (Jin, Ismail, Muharam, & Alias, 2021), a point-based accuracy assessment was conducted using Google Earth as a verification source.

Madrigal-Martínez, S., & Miralles García, J. L. (2019). Land-change dynamics and ecosystem service trends across the central high-Andean Puna. *Scientific Reports*, *9*(1), 1–12. https://doi.org/10.1038/s41598-019-46205-9

Gergel, S. E., & Turner, M. G. (Eds.). (2017). *Learning landscape ecology: a practical guide to concepts and techniques*. Springer.

Jin, D. H., Ismail, M. H., Muharam, F. M., & Alias, M. A. (2021). Evaluating the impacts of land use/land cover changes across topography against land surface temperature in Cameron Highlands. *PLoS ONE*, *16*(5 May), 1–26. https://doi.org/10.1371/journal.pone.0252111

*What spectral vegetation index did use to extract plant characteristics and classify them?*

Spectral vegetation indexes were not used by the project team from MAE and MAGAP for the Land Use Land Cover classification, instead, a supervised process with RBD signatures for LULC classes in the ENVI software. See our two first responses in section A for a further explanation.

*The scale of maps, including MAE maps and the maps taken from Landsat images analysis, is unknown.*

Completed, This is detailed in the revised methods. Resulting maps were produced in a mapping scale of 1:100,000 with Landsat (TM) images as main inputs.

***B. The role of stimuli in the spotted changes is not thoroughly covered with regard to their relationship with each other.***

*The results of decreasing the dimensions are not thoroughly highlighted and elaborated for the five mentioned groups.*

Completed. Following the reviewer’s comment, we have added in the Methods an explanation of the results from the dimension reduction after conducting the PCAs within each grouping driver.

*The authors could use Markov's advanced mode known as Latent MC for use in the real world.*

We did not use Latent Markov Models, which is a very interesting conceptual and methodological approach to explore ecological systems. However, we used the transition probabilities obtained in Markov chain analyses and integrate them into another very powerful statistical model (General Additive Model), currently in use by ecologists (Woods 2017a), to reveal important drivers of change for land use land cover dynamics. In that sense, we consider it as an alternative to LMM and an innovative inferential method that seek to uncover the relationships between factors driving dynamics in ecological systems and thereby predict them in quantitative terms.

*As a data screening model, the results of the DPSIR model are not expressed for all variables (drivers) and are not complete.*

In response to this comment, we included a graphical scheme (new Fig 1) and a detailed description of our proposed conceptual framework in the Method’s section. Afterwards, we clearly described the way we implemented the DPSIR framework, explaining how we are conceiving the driving forces and their effect on LULC transitions. Subsequently, we described the analytical approach to include different drivers of change within groups, the way we conducted variable screening and the dimension reduction process, to finally present the statistical model that we used to test the driving forces that explain LULC changes in our case study.

*Why is DPSIR listed in the introduction?*

Since we are proposing an adapted DPSIR approach for tropical mountain systems and we implemented an initial assessment using this approach in a case study in the highlands of Ecuador, we consider important to expand the description of the DPSIR approach in the Introduction.

For a more detailed explanation of the proposed conceptual framework, please see our response to the second reviewer's first comment.

*What is the evaluation and policy method that led to the selection of 13 variables for LULC? Not exactly specified.*

In the Methods we added a detailed explanation of the criteria used for selecting the variables included in the model to explain LULC change, and the way we fulfilled each selection criteria.

Briefly, we followed the criteria selection in the context of a DPSIR framework, as proposed by: Wang Z, Zhou J, Loaiciga H, Guo H, Hong S. A DPSIR model for ecological security assessment through indicator screening: A case study at Dianchi Lake in China. PLoS One. 2015;10(6):1–13. These authors, propose the following criteria: ‘(1) Relevancy: indicators should reflect the underlying cause of environmental change. (2) Availability: the indicator data should be available, accessible, and consistent within the period of analysis. (3) Independence: indicators must be independent of each other to eliminate multicollinearity. (4) Representativeness: each indicator used in the model must represent a category or phenomenon of its own, and must provide superior information to other indicators in a similar category’ (p.4).

***C. The article deals with data screening (ecological and socio-economic variables), evaluation, decision-making, monitoring, and policy-making, but the content is scattered, inadequate and unreliable. Each of these topics should be defined separately in its section and the results of each.
Any decision in data mining process shall be based on the type of data and primary knowledge of the data structure and behavioral pattern. This is not observed in any of the statistical methods used.***

As mentioned in our previous response, our updated manuscript provides an improved explanation of the connections between the Drivers of change and the actual pressures on the environment in the context of the adapted DPSIR framework. We consider that our current explanation of the framework set the stage to better understand the methods and the results presented in our article. Here, we proposed an adaptation of the whole DPSIR framework in the context of tropical mountain systems, with all the 5 elements of the conceptual model.

We also clarified that the purpose of the current paper is to develop an initial ecosystem assessment including only Drivers and Pressures. A further ecosystem assessment, to complete the assessment with all 5 elements of the DPSIR, is under work in our case study.

Additionally, we added a section to describe the results of our data screening and the dimension reduction after conducting Principal Component Analysis (PCAs), in each group of the tested drivers of change.

*The statistical methods are outdated and the submitted study lacks novelty.*

We consider that our study is unique in different aspects. Firstly, it adapts the DPSIR holistic approach to the context of tropical mountain systems and implement the first elements of the framework in a sensitive region of the northeastern Ecuadorian Andes. Then, it contributes to identify the key characteristics of tropical mountain social-ecological systems that should be represented in ecosystem assessments to support evidence-based policy and management actions. We connected our proposed conceptual framework with a step by step methodological approach, which was initially implemented in a case study located in the highlands of northern Ecuador

Secondly, we portrayed land use land cover (LULC) dynamics using Markov-chain probabilities by elevation and geographic settings, which is an interesting characterization, recently conducted in other regions but limited for the highland landscapes in the Andes. In addition, we consider that Markov chain analysis is a well-known approach to describe LULC transitions, currently in use as supported by the following recent publications:

Gergel, S. E., & Turner, M. G. (Eds.). (2017). *Learning landscape ecology: a practical guide to concepts and techniques*. Springer.

Hamad, R., Balzter, H., & Kolo, K. (2018). Predicting land use/land cover changes using a CA-Markov model under two different scenarios. *Sustainability (Switzerland)*, *10*(10), 1–23. https://doi.org/10.3390/su10103421

Kumar, S., Radhakrishnan, N., & Mathew, S. (2014). Land use change modelling using a Markov model and remote sensing. *Geomatics, Natural Hazards and Risk*, *5*(2), 145–156. https://doi.org/10.1080/19475705.2013.795502

Liping, C., Yujun, S., & Saeed, S. (2018). Monitoring and predicting land use and land cover changes using remote sensing and GIS techniques—A case study of a hilly area, Jiangle, China. *PLoS ONE*, *13*(7), 1–23. https://doi.org/10.1371/journal.pone.0200493

Finally, we integrated Markov’s transitions probabilities (as a response variable) with a variety of freely available geospatial and temporal data into Generalized Additive Models (GAMs) to uncover the factors driving such landscape dynamics in a sensitive region of the northern Ecuadorian Andes. We used GAM regressions to elucidate two types of transitions estimated through Markov chain analysis: 1) the probability of natural ecosystems loss, and 2) the probability to change towards anthropic environments.

GAMs are very powerful statistical models, used extensively in environmental modelling and provide great scope to model complex relationships between covariates, as is exemplified in the following references:

Liping, C., Yujun, S., & Saeed, S. (2018). Monitoring and predicting land use and land cover changes using remote sensing and GIS techniques—A case study of a hilly area, Jiangle, China. *PLoS ONE*, *13*(7), 1–23. https://doi.org/10.1371/journal.pone.0200493

Wood, S. (2017). *Generalized Additive Models: An Introduction with R.* (2nd ed.). CRC Press.

*Methodology needs major improvement.*

Based on different suggestions and inquiries from all the reviewers, we have improved our text in the Methodology to better explain all the steps that we conducted to fulfill our objectives. We consider that this section from the updated version of the manuscript has largely improved.

**Specific comments from Reviewer #2:**

*1. Theoretical Framework: No explicit theoretical framework and important theoretical assumptions is observed?*

As mentioned in our response to comment B from the first reviewer, we are proposing an adapted Driver Pressure State Impact Response (DPSIR) framework for tropical mountain systems, and it was not fully developed before. Therefore, for our updated manuscript we have highlighted this contribution in the Introduction section. Further, we described that for the purpose of the current article we are developing an initial ecosystem assessment in the context of the proposed DPSIR, to uncover the driving forces that exert pressure on the landscapes, by examining LULC changes. In the Methods, we included a graphical scheme (new Fig 1) and a detailed description of our proposed conceptual framework. Afterwards, we clearly described the way we implemented the DPSIR framework, explaining how we are conceiving the driving forces and their effect on LULC transitions. Subsequently, we described the analytical approach to include different drivers of change within groups, the way we conducting variables screening and the dimension reduction process, and present the statistical model that we used to test the driving forces that explain LULC changes in our case study.

*2. Concepts: A central concept of the study is not adequately and clearly defined? No new concepts have been added to the discipline.*

Completed. We have rewritten much of the introduction to include and highlight key concepts to describe the issues and focus of the article (eg. land use, land cover, ecosystem services).

*3. Argument: Central argument is missing? It must be tightly and well written with examples referring from global, regional and local levels. Lots of studies have been published in recent years on a similar theme.*

We have rewritten the Introduction to better present the central argument, supported by more recent publications

*4. Literature review and use of references are inadequate. Most of the references are older than 10 years and only a few latest references are cited. Some recently updated references need to be added. Following latest references may also be cited in the manuscript for value addition:
• Mishra, P.K. Rai, A. and Rai, S.C. (2020) Land Use and Land Cover Change Detection using Geospatial Techniques in Sikkim Himalaya, India. Egyptian Journal of Remote Sensing and Space Sciences. 23 (2):133-143. doi.org/10.1016/j.ejrs.2019.02.001 IF: 5.18 (2020).

• Mishra, P.K. and Rai, A. (2020) Role of Unmanned Aerial Systems for Natural Resource Management. Journal of the Indian Society of Remote Sensing. ISSN 0974-3006. DOI: 10.1007/s12524-020-01230-4 (SCOPUS). IF: 1.56 (2020).*

We have carefully reviewed and updated our references.

*5. The title is vague and too lengthy.*

We have revised the title to better summarise the focus of the article

*6. This study does not provide any new contribution in the field. The study lacks novelty and continuity in the analysis and provides general findings.*

We consider that our study is unique in different aspects. We presented a detailed response to similar issues in our response to comment C from the first reviewer. Please refer to this previous section.

*7. Authors have selected “Official Land Use Land Cover (LULC) maps from the Ministry of Environment of Ecuador (MAE) of four periods of time:* [*1990, 2000, 2008*](callto:1990,%202000,%202008) *and 2014”. However, the months and percentage of cloud cover available in satellite data are not mentioned.*

Completed. We have added a detailed description of the classification methods conducted by the project team from MAE and MAGAP to produce the official LULC maps. We explained that to optimize the use of cloud-free satellite images to generate the coverage map for each year, a range of 12 months before and after the defined year was considered

*8. While conducting LULC analysis, accuracy assessments play a major role in determining the overall accuracy of results. In the present manuscript accuracy assessments of maps taken from the Ministry of Environment of Ecuador is not mentioned.*

*Completed*. We have added an explanation in the Methods section. The accuracy assessment analysis implemented an independent interpretation process, where an experienced image expert classified the coverage for each sample obtained from a stratified random sampling protocol, according to the methods proposed in the FRA global remote sensing survey (Forestry Department, 2009); this was done based on the Level 1 legend of the LULC maps of Continental Ecuador. A confusion matrix was created using the JRC Validation Tool program for the sampling areas (Simonetti et al., 2011), and the following overall accuracy values were obtained: 69%, 73%, 76% and 85% for the years 1990, 2000, 2008 and 2014, respectively (MAE, 2016).

*9. Accuracy assessment of 5 additional topologies produced by authors is also not available in the manuscript.*

We added and explanation of our accuracy assessment in the Methodology as follows:

Following the methods proposed by (Jin et al., 2021), a point-based accuracy assessment was conducted using Google Earth as a verification source. After that, a confusion matrix was created using 600 random points obtained from a stratify sampling scheme over the altitudinal bands. The resulting overall accuracy of the edited maps ranged from 82 to 86%. The editing process, using visual digitalization, over the LULC official vector layers from the periods of interest and the accuracy assessment were conducted in QGIS 3.10 (QGIS Development Team., n.d.)

Jin, D. H., Ismail, M. H., Muharam, F. M., & Alias, M. A. (2021). Evaluating the impacts of land use/land cover changes across topography against land surface temperature in Cameron Highlands. *PLoS ONE*, *16*(5 May), 1–26.

*10. Authors have mentioned in lines 124-125 that they digitized these 5 topologies that include planted forests, developed areas (populated zones), horticulture (areas represented by greenhouses) and natural water bodies. Line 138 authors stated that “because the study area corresponds to the major centre of floriculture production for the export market in 138 Ecuador [37,38], we added floriculture crop, as a separate typology from the agricultural land”. Areas represented by greenhouses can also have vegetations and other crops in them how was the area under floriculture estimated?*

We have clarified and corrected the main text. The previous manuscript was incorrect in describing that the floriculture crop as a separate typology from the agricultural land typology; instead in the LULC official maps, it was included as part of the infrastructure, within the developed areas. We corrected the text and added some more detail on the characteristics of floriculture production.

We also expanded the justification to assume that greenhouses in our study region were considered for floriculture production

*11. Digitization requires expertise in image interpretation and classes such as floriculture or horticulture are hard to map on Landsat TM data. And using Greenhouses as an indicator of horticulture and mapping entire areas of greenhouses as horticulture or floriculture without surveying is not recommended as many of them might not be in use. Results of LULC show that there is a huge growth in floriculture in the study area. However, digitizing greenhouse as floriculture is not a reliable or accurate method of mapping.*

We have clarified and expanded the description of our editing process, where we used different sources to improve the classification such as the floriculture cadastral map from the geoportal of the Ministry of Agriculture.

Additionally, it is important to point out that a field study, complementary to this landscape analysis is being conducted by the main author, to understand the effect of land use on biodiversity and ecosystem services, therefore, regular field visits have been carried out, providing the author a good geographic understanding of the current situation of the study area

*12. No Field data/assessment is available for produced LULC maps.*

We have described in the Methods the assessment conducted by MAE and MAGAP to produce the official LULC maps. A similar inquiry was posed by the first reviewer. Please see our detailed response for the second comment of reviewer #1.

In addition, a point-based accuracy assessment was conducted using the historical images from Google Earth as a verification source for the final maps obtained after our editing process in our study region. This is an alternative method to field data verification and assessment as proposed by: Jin, D. H., Ismail, M. H., Muharam, F. M., & Alias, M. A. (2021). Evaluating the impacts of land use/land cover changes across topography against land surface temperature in Cameron Highlands. *PLoS ONE*, *16*(5 May), 1–26.

*13. Four periods of time:* [*1990, 2000, 2008*](callto:1990,%202000,%202008) *and 2014 have been selected in the manuscript. From 2014 to 2021 the world has seen exponential growth in terms of LULC changes. Why not produce LULC maps of 2020 or 2021 and then do the analysis.*

We decided to use official LULC maps and other freely available databases from distinct sources (demographic, climatic, topographic, etc.) as a practical DPSIR implementation example that could be replicated to understand environmental change (LULC transitions and their drivers) in a context of data scarcity and low technical capacities for the processing of remote sensing information required for land management and planning. This corresponds to the reality at distinct territorial level of governance in developing countries.

For our LULC analysis we only included the four periods of time [1990, 2000, 2008](callto:1990,%202000,%202008) and 2014 from which available and comparable maps were available when the analysis took place

*14. If the selected data is almost 7 years old, how do the authors think their results related to LULC change till 2014 and drivers of change are relevant today?*

We consider the LULC trends observed in our landscape territory up to 2014 provides a recent characterization of the trajectories that are occurring in northern Ecuador. We included information until this year of analysis because it was available when the study was conducted

*15. The whole manuscript is consisting of multiple grammatical and typographical errors.*

Completed. As mentioned before. We already overcome this problem. The manuscript was revised by English native speakers from an editing and proofreading service.

*16. Data sets chosen for the study has a different spatial resolution for both data. Landsat 5 (30m) and LISS 3 (23.5m). Rescaling of the data was not mentioned in the manuscript. Not rescaling both datasets on the same resolution causes inaccuracy in change detection due to different pixel sizes and even if it’s done the gap of resolution between two original data sets should be minimum. Here rescaling data from 30 m to 23.5m or vice versa would cause serious errors/loss of data.*

The classification analysis performed by the team of geographers from MAE and MAGAP used primarily LANDSAT images from different years at a spatial resolution of 30m.

LISS satellite images were not used

*17. Limitations of the study are not mentioned anywhere e.g., areas and reasons for misclassification.*

Following the suggestion from the reviewer, we have expanded the limitation section in our discussion. We expanded the methodological limitations that could led to misclassification of the LULC maps

*18. References have to be rechecked as they lack similarity in writing style. In a few references, the page number is written confusingly.*

Completed. We have carefully checked the references throughout the text and in the list of references to fulfill the PLOS ONE style.

*19. The practical implacability of the study in any other field of work is missing. Recommendations for Future Work may add value to the manuscript.*

Expand the recommendations focusing in the implacability of our findings.

*20. The authors must check the grammar, consistency and flow of the texts in the manuscript before submitting for publication.*

Completed. The manuscript was revised by English native speakers from an editing and proofreading service on language improvement

**Specific comments from Reviewer #3:**

*1. The analysis has been performed between 1990-2014. Unless there is a very good reason, I would expect to see the analysis to the latest time.*

As it was explained in the comment N. 13 from Reviewer N.2, we decided to use official LULC maps and other freely available databases from distinct sources (demographic, climatic, topographic, etc.) as a practical DPSIR implementation example that could be replicated to understand environmental change (LULC transitions and their drivers) in tropical mountain systems. These regions, mostly situated in developing countries, are characterized by a context of data scarcity and low technical capacities for the processing of remote sensing information required for land management and planning.

Specifically, this situation depicts the situation from our study region.

For our LULC analysis we only included the four periods of time [1990, 2000, 2008](callto:1990,%202000,%202008) and 2014 from which available and comparable maps were available when the analysis took place.

*2. L 174-177 Kindly make the explanation and the table 1 consistent*

Completed. We have made all the terminology of the driving forces consistent between the text and Table 1. Additionally, we added a description of the variables included within each grouping driver.

*3. There are two tables labelled Table 2 ( L237 and L 320). The text nowhere explains table 3 even if the later is labelled as table 3.*

Completed. We have corrected the error.

**Specific comments from Reviewer #4:**

*1) Modify the abstract and add some content showing the methodology applied and the clear results obtained from the study. The conclusion part of the abstract is redundant and provides too general a description of results.*

Completed. Accordingly with the reviewer’s suggestion, the contextual and methodological framework was added to the abstract. We have also synthesized the conclusion and provided more specific results.

*2) The paper has many long sentences, grammar errors and is poorly written. Extensive English editing is needed.*

Completed. We already overcome this problem. The manuscript was revised by English native speakers from an editing and proofreading service.

*3) The Introduction section should be improved, and at the end of the introduction, you should clearly state the objective. For instance, in your last statement in the introduction, you have stated that you will employ the DPSIR framework in assessing the role of different drivers on LULCC in the study area, but it is not featured out in your methodology. Please clarify this.*

Completed.. In the revised introduction we have better explained the DPSIR approach, as our guiding conceptual framework, which connects Drivers and Pressures in a landscape pattern analysis, additionally the DPSIR framework was properly operationalized in the methods.

We further elaborated on this subject on comment

*(4) In the results section, merge heading “Coverage area for each year” and “Land-change dynamics through time”*

Completed. We included the reviewer´s suggestion

*(5) In Table 2. Rename the caption to match the content of the paper*

Completed. We have corrected the error

*(6) [318]- Renumber the table, is not Table 2 as it is, I think it should refer to Table 3*

Completed. We have corrected the error

*(7) [363-366]- not clear what you wanted to say*

Completed. We have rewritten the paragraph to clarify the main point.

*8) [392-389]- too long sentence not clear what you wanted to say*

We have edited this paragraph following the reviewer’s suggestion.

*(9) [457-462]- English should be checked*

As mentioned elsewhere. We have corrected this problem with the aid of an Editing service for proofreading our revised draft.

10) [520-526]- too long sentence not clear what you wanted to say

We have edited this paragraph following the reviewer’s suggestion.

We look forward to hearing from you in due time regarding our submission and to respond to any further questions and comments you may have.

Sincerely,

Paulina Guarderas, M. Sc.

Corresponding Author

| Facultad de Ciencias Biológicas,  Universidad Central del Ecuador  Numa Pompilio Liona S/N y Yaguachi,  QUITO, Ecuador  E-mail: apguarderas@uce.edu.ec  Cel: 593 992403093 | Additional Contact:  Biodiversity and Landscape Unit,  Gembloux Agro Bio-Tech,  University of Liège  Passage des Dport s 2, 5030  GEMBLOUX, Belgique  E-mail: ap.guarderas@doct.uliege.be |
| --- | --- |
