# Supplementary material for: Land use and land cover change in a tropical mountain landscape of northern Ecuador: Altitudinal patterns and driving forces
Source: PLoS One. 2022 Jul 27;17(7):e0260191. doi: 10.1371/journal.pone.0260191 (PMC9330684; doi:10.1371/journal.pone.0260191)
Supplement: Attachment — Submitted filename: Comments PONE.docx [file pone.0260191.s013.docx]

Dear Editor

I have reviewed the manuscript number PONE-D-21-35047 full title ‘Land use and land cover change in a tropical mountain landscape of northern Ecuador: altitudinal patterns and driving forces’. The revised manuscript is improved in quality and readability. On the other hand, I still have few suggestions author/s should take into consideration:

1. Title should be *‘Land use and land cover change in a tropical mountain landscape of northern Ecuador’.*
2. *Abstract:* Generalized Additive Model (GAM) (Line 32) and Multiregression models (GAM) (Line 41) is creating confusion. Please use different abbreviation for two models.
3. Suggested references are missing in the revised manuscript reference section.

- Mishra, P.K. Rai, A. and Rai, S.C. (2020) Land Use and Land Cover Change Detection using Geospatial Techniques in Sikkim Himalaya, India. ***Egyptian Journal of Remote Sensing and Space Sciences.*** 23 (2):133-143. doi.org/10.1016/j.ejrs.2019.02.001 IF: 5.18 (2020).
- Mishra, P.K. and Rai, A. (2020) Role of Unmanned Aerial Systems for Natural Resource Management. ***Journal of the Indian Society of Remote Sensing***. ISSN 0974-3006. DOI: 10.1007/s12524-020-01230-4 (SCOPUS). IF: 1.56 (2020).

4. The practical implacability of the study in any other field of work is missing. Recommendations for Future Work may add value to the manuscript.

Best Wishes!

Dr. Prabuddh K Mishra
