# Supplementary material for: Land use and land cover change in a tropical mountain landscape of northern Ecuador: Altitudinal patterns and driving forces
Source: PLoS One. 2022 Jul 27;17(7):e0260191. doi: 10.1371/journal.pone.0260191 (PMC9330684; doi:10.1371/journal.pone.0260191)
Supplement: Attachment — Submitted filename: Response to Reviewers.docx [file pone.0260191.s015.docx]

April 28, 2022

Dear Dr. Manoj Kumar
Academic Editor
PLOS ONE

Thank you for the opportunity to resubmit a revised version of the manuscript PONE-D-21-35047R1, titled “Land use and land cover change in a tropical mountain landscape of northern Ecuador: altitudinal patterns and driving forces“ to PLOS ONE

My co-authors and I have read in detail your email and all the comments from the reviewers, and when specific suggestions were included we have been able to incorporate changes in the manuscript, which have been highlighted in the manuscript. In contrast, for other general comments we have explained our responses in the rebuttal letter.

Here is a point-by-point response to your suggestions and to the reviewers’ comments and concerns.

**Responses to the Academic Editor’s suggestions:**

*1. Journal Requirements:

Please review your reference list to ensure that it is complete and correct. If you have cited papers that have been retracted, please include the rationale for doing so in the manuscript text, or remove these references and replace them with relevant current references. Any changes to the reference list should be mentioned in the rebuttal letter that accompanies your revised manuscript. If you need to cite a retracted article, indicate the article’s retracted status in the References list and also include a citation and full reference for the retraction notice.*

Completed. Accordingly with your suggestion, we have carefully reviewed the reference list and made some adjustments described as follow:

We correctly placed the references n. 20 and 21 as follow:

Reference n. 20: *Brandt JS, Townsend PA. Land use - Land cover conversion, regeneration and degradation in the high elevation Bolivian Andes. Landsc Ecol. 2006;21(4):607–23*)

was moved to line 84.

The new reference n. 21: *Vanacker V, Molina A, Torres R, Calderon E, Cadilhac L. Challenges for research on global change in mainland Ecuador. Neotrop Biodivers [Internet]. 2018;4(1):114–8. Available from: doi: 10.1080/23766808.2018.1491706 was added to line 87* was moved to line 87.

Based on one suggestion from the Reviewer#2, we added the reference n.78 in the Discussion (lines 578, 714 and 719):

*Mishra, P.K. Rai, A. and Rai, S.C. (2020) Land Use and Land Cover Change Detection using Geospatial Techniques in Sikkim Himalaya, India. Egyptian Journal of Remote Sensing and Space Sciences. 23 (2):133-143. doi.org/10.1016/j.ejrs.2019.02.001*

We added the reference n.90 in the discussion section, since it demonstrates the importance of using Google Earth images and tools for detecting land use changes:

*Damtea W, Kim D, Im S. Spatiotemporal analysis of land cover changes in the chemoga basin, Ethiopia, using Landsat and google earth images. Sustain. 2020;12(9).*

**Responses to the reviewer’s general observation and questions**

1. If the authors have adequately addressed your comments raised in a previous round of review and you feel that this manuscript is now acceptable for publication, you may indicate that here to bypass the “Comments to the Author” section, enter your conflict of interest statement in the “Confidential to Editor” section, and submit your "Accept" recommendation.

Reviewer #1: All comments have been addressed

Reviewer #2: All comments have been addressed

Reviewer #3: All comments have been addressed

Reviewer #4: All comments have been addressed

Since the reviewers agreed that all comments have been addressed, we have nothing else to add.

2. Is the manuscript technically sound, and do the data support the conclusions?

The manuscript must describe a technically sound piece of scientific research with data that supports the conclusions. Experiments must have been conducted rigorously, with appropriate controls, replication, and sample sizes. The conclusions must be drawn appropriately based on the data presented.

Reviewer #1: Partly

Reviewer #2: Yes

Reviewer #3: Yes

Reviewer #4: Partly

We answered in detailed all the remarks made by the Reviewer#1 in the following section.

In addition, we have accepted most of the suggestions from the Reviewer#4, which were included in the document uploaded as attachment; however, they were not substantive changes.

Finally, we consider that with the changes made on the previous revised version, we have improved the theoretical and the methodological framework of the manuscript to better connect all the elements of our study, fulfilling this evaluation criterion.

3. Has the statistical analysis been performed appropriately and rigorously?

Reviewer #1: N/A

Reviewer #2: Yes

Reviewer #3: Yes

Reviewer #4: Yes

We have nothing to add to this criterion.

4. Have the authors made all data underlying the findings in their manuscript fully available?

The [PLOS Data policy](http://www.plosone.org/static/policies.action#sharing) requires authors to make all data underlying the findings described in their manuscript fully available without restriction, with rare exception (please refer to the Data Availability Statement in the manuscript PDF file). The data should be provided as part of the manuscript or its supporting information, or deposited to a public repository. For example, in addition to summary statistics, the data points behind means, medians and variance measures should be available. If there are restrictions on publicly sharing data—e.g. participant privacy or use of data from a third party—those must be specified.

Reviewer #1: Yes

Reviewer #2: Yes

Reviewer #3: Yes

Reviewer #4: Yes

As it is recognized by all the reviewers, we have made all our data fully available in the Zenodo’s open access repository. This is the DOI 10.5281/zenodo.5911876 to access our datasets.

5. Is the manuscript presented in an intelligible fashion and written in standard English?

PLOS ONE does not copyedit accepted manuscripts, so the language in submitted articles must be clear, correct, and unambiguous. Any typographical or grammatical errors should be corrected at revision, so please note any specific errors here.

Reviewer #1: Yes

Reviewer #2: Yes

Reviewer #3: Yes

Reviewer #4: Yes

We are very pleased that all reviewers agreed that the English grammar of the manuscript has improved significantly

**Responses to Specific comments from different reviewers:**

**Specific comments from Reviewer #1:**

After reviewing the revised file submitted by the authors, compliance with the requirements in the initial review phase is generally approved. However, the following details have been received less attention.
Remote sensing experts believe that accuracy of over 85% is acceptable for detecting changes in satellite imagery. Hene, reputable papers from remote sensing experts should be studied in this regard. Assuming that the number and dispersion of sampling points (Rois) were observed, the accuracy of the article is not satisfactory. However, with a little leeway, the accuracy of 69-73 and 79 can be accepted.

As we explained in our previous rebuttal letter, to refine the results and obtain a more accurate map of the study area, a thorough editing process of the official vector maps from the different study periods was carried out. To support this editing process, as carried out by (Madrigal-Martínez and Miralles i García 2019), distinct secondary sources of information were revised such as field points, Google Earth images, orthophotographs and other official such as the ecosystem coverage (<http://ide.ambiente.gob.ec/mapainteractivo/>), the floriculture cadastral and other maps from the Ministry of Agriculture (<http://geoportal.agricultura.gob.ec/>). In addition, composite LANDSAT images from our study area, using radiometric enhancements, three-dimensional visualization and spectral band combinations were also used through Google Earth Engine (Gorelick et al. 2017). From the editing process, mainly five typologies were improved. These included: planted forests, developed areas (populated zones), floriculture (areas represented by greenhouses) and natural water bodies. Following the methods proposed by (Jin et al. 2021), a point-based accuracy assessment was conducted using Google Earth as a verification source.

In the previous version of our manuscript, we added and explanation of our accuracy assessment in the Methodology as follows:

Following the methods proposed by (Jin et al. 2021), a point-based accuracy assessment was conducted using Google Earth as a verification source. After that, a confusion matrix was created using 600 random points obtained from a stratify sampling scheme over the altitudinal bands.

**The resulting overall accuracy of the edited maps** **ranged from 82 to 86%;** therefore, we consider we have improved the accuracy to detect changes in our study area close to the accuracy thresholds suggested by remote sensing experts, as stated by the Reviewer.

To study the plant and agricultural species, a scale of 1.100000 is small. This scale is more suitable for maps obtained from low-resolution satellite images and for studying the regional climate.

The difficulties to detect similar spectral reflectance patterns between neighboring landscape segments could be overcome by using Google Earth images in a post-classification process (Damtea, Kim, and Im 2020). In our analysis, discriminating natural from plantation forests (which are encompassed by different plant species) could have some of these difficulties. We, therefore, used the editing or post-classification process described above, using different sources and the powerful options provided in the Google Earth Engine to improve the identification of unclear objects (Midekisa et al. 2017; Sidhu, Pebesma, and Câmara 2018).

Google Earth is not a good source for specific remote sensing tasks. It is more reliable to use mapping organization maps, aerial mapping, or field fraternity mapping operations.

We also consider that ground field surveys are preferable to remote sensing data (such as Google Earth) for accuracy detection or supervised classification uses. However, recent articles are demonstrating the practicality of using Google Earth (GE) images when other sources are not available. GE offers the possibility of conducting LULC analysis with high to medium spatial resolution and with a multitemporal scope (Damtea, Kim, and Im 2020).

Damtea, Wubeshet, Dongyeob Kim, and Sangjun Im. 2020. “Spatiotemporal Analysis of Land Cover Changes in the Chemoga Basin, Ethiopia, Using Landsat and Google Earth Images.” *Sustainability (Switzerland)* 12(9).

Gorelick, Noel et al. 2017. “Google Earth Engine: Planetary-Scale Geospatial Analysis for Everyone.” *Remote Sensing of Environment* 202(2016): 18–27. doi: 10.1016/j.rse.2017.06.031.

Jin, Darren How, Mohd Hasmadi Ismail, Farrah Melissa Muharam, and Mohamad Azani Alias. 2021. “Evaluating the Impacts of Land Use/Land Cover Changes across Topography against Land Surface Temperature in Cameron Highlands.” *PLoS ONE* 16(5 May): 1–26. doi: 10.1371/journal.pone.0252111.

Madrigal-Martínez, Santiago, and José Luis Miralles i García. 2019. “Land-Change Dynamics and Ecosystem Service Trends across the Central High-Andean Puna.” *Scientific Reports* 9(1): 1–12.

Midekisa, Alemayehu et al. 2017. “Mapping Land Cover Change over Continental Africa Using Landsat and Google Earth Engine Cloud Computing.” *PLoS ONE* 12(9): 1–15.

Sidhu, Nanki, Edzer Pebesma, and Gilberto Câmara. 2018. “Using Google Earth Engine to Detect Land Cover Change: Singapore as a Use Case.” *European Journal of Remote Sensing* 51(1): 486–500. https://doi.org/10.1080/22797254.2018.1451782.

**Specific comments from Reviewer #2:**

I have reviewed the manuscript number PONE-D-21-35047 full title ‘Land use and land cover change in a tropical mountain landscape of northern Ecuador: altitudinal patterns and driving forces’. The revised manuscript is improved in quality and readability. On the other hand, I still have few suggestions author/s should take into consideration:
1. Title should be ‘Land use and land cover change in a tropical mountain landscape of northern Ecuador’.

We thank the reviewer for this suggestion; however, we prefer the longer title because we feel that it better describes its content and scope.

2. Abstract: Generalized Additive Model (GAM) (Line 32) and Multiregression models (GAM) (Line 41) is creating confusion. Please use different abbreviation for two models.

Completed. As we are referring to the same model in line 32 and line 41 in the Abstract, we have used the same abbreviation to avoid confusion.

3. Suggested references are missing in the revised manuscript reference section kindly add for value adition.
• Mishra, P.K. Rai, A. and Rai, S.C. (2020) Land Use and Land Cover Change Detection using Geospatial Techniques in Sikkim Himalaya, India. Egyptian Journal of Remote Sensing and Space Sciences. 23 (2):133-143. doi.org/10.1016/j.ejrs.2019.02.001
• Mishra, P.K. and Rai, A. (2020) Role of Unmanned Aerial Systems for Natural Resource Management. Journal of the Indian Society of Remote Sensing. ISSN 0974-3006. DOI: 10.1007/s12524-020-01230-4

We have complemented our references with the first suggested article. However, we feel that the second one does not relate to the main methodology used in our manuscript, therefore, we did not included it.

4. The practical implacability of the study in any other field of work is still missing. Recommendations for Future Work may add value to the manuscript.

Best Wishes!

We don’t fully understand this suggestion because our revised manuscript already includes a section for implications (from line 691 to 721); however, we added another sentence to highlight the implication of our findings for conserving native ecosystems (lines 716-717).

We deleted the subtitle of limitations because the last paragraphs before the conclusions described the implications of our study.

Finally, at the end of the conclusion section, we added our ideas for future work.

Reviewer #3: The authors have provide enough explanation to my concern. I still wished they had the latest year covered in the analysis but I do not seek that as the sole reason to reject it. Hence, I recommend for acceptance to publish provided that it meets other publication criteria as per the guideline ( copy right and others).

We thank the reviewer#3 for recommending the acceptance of our manuscript in PLOS ONE.

As we explained in our previous rebuttal letter. We decided to use official LULC maps and other freely available databases from distinct sources (demographic, climatic, topographic, etc.) as a practical DPSIR implementation example that could be replicated to understand environmental change (LULC transitions and their drivers) in tropical mountain systems. These regions, mostly situated in developing countries, are characterized by a context of data scarcity and low technical capacities for the processing of remote sensing information required for land management and planning.

Specifically, this situation depicts the situation from our study region.

For our LULC analysis we only included the four periods of time [1990, 2000, 2008](callto:1990,%202000,%202008) and 2014 from which available and comparable maps were available when the analysis took place.

Reviewer #4: Dear Authors,
Thank you for your manuscript “Land use and land cover change in a tropical mountain landscape of northern Ecuador: altitudinal patterns and driving forces”. The title is now well written covering the content of the paper. I recommended accepting the topic as it fully matches the scope of the journal “PLOS ONE”. Also, now authors have incorporated most reviewers’ comments provided to them making the manuscript much better. But there are some issues that need to be addressed by the authors, see the attached track change word document.

We thank the reviewer#4 for recommending the acceptance of our manuscript in PLOS ONE.

We have accepted most of the suggestions made by the reviewer in the manuscript, they were mainly changes in rephrasing or shortening the sentences. Only one suggestion was not accepted, it was the deletion of S2Fig; we consider important to keep it as it is, since it complements the analysis of native ecosystems in different elevation bands. We rewrote the legend title of S1Fig and S2Fig.

We look forward to hearing from you in due time regarding our submission and to respond to any further questions and comments you may have.

Sincerely,

Paulina Guarderas, M. Sc.

Corresponding Author

| Facultad de Ciencias Biológicas,  Universidad Central del Ecuador  Numa Pompilio Liona S/N y Yaguachi,  QUITO, Ecuador  E-mail: apguarderas@uce.edu.ec  Cel: 593 992403093 | Additional Contact:  Biodiversity and Landscape Unit,  Gembloux Agro Bio-Tech,  University of Liège  Passage des Dport s 2, 5030  GEMBLOUX, Belgique  E-mail: ap.guarderas@doct.uliege.be |
| --- | --- |
