# Supplementary material for: Land use and land cover change in a tropical mountain landscape of northern Ecuador: Altitudinal patterns and driving forces
Source: PLoS One. 2022 Jul 27;17(7):e0260191. doi: 10.1371/journal.pone.0260191 (PMC9330684; doi:10.1371/journal.pone.0260191)
Supplement: Attachment — Submitted filename: Response to Reviewers.docx [file pone.0260191.s016.docx]

June 14, 2022

Dear Dr. Manoj Kumar
Academic Editor
PLOS ONE

Thank you for the opportunity to resubmit a revised version of the manuscript PONE-D-21-35047R1, titled “Land use and land cover change in a tropical mountain landscape of northern Ecuador: altitudinal patterns and driving forces“ to PLOS ONE.

Since the reviewers raised no further observations or comments on the latest version of the manuscript, my co-authors and I have concentrated on thoroughly revising the citations and bibliographic references so that they comply with the citation style of the journal and contain complete information, as it was requested in your last message.

Likewise, we have reviewed in detail which references have been retracted or replaced in the manuscript text and the Reference list to provide the rationale for doing so.

In fact, 15 references that were included in the original manuscript no longer appear in the most recent version. Before resubmitting the manuscript, we decided to revise the bibliography because we considered that it was very extensive and some references were not as up to date, in addition, we found that some references were redundant or could be incorporated in review, synthesis or regional analysis articles. We also found errors in the list of references where some citations were repeated because they were uploaded twice, with small differences, in the Reference Management Software that we used.

Below is a list of the references that were included in the first draft of the manuscript and are no longer in the latest version, in addition, we added an explanation for retracting or replacing the references.

| **Retracted references in the first draft** | | **Current references in the last draft** | | **Explanation for retracting or replacing the references** |
| --- | --- | --- | --- | --- |
| **N.** | **References** | **N.** | **References** |  |
| 1 | Körner C, Ohsawa M, Spehn E, Berge E, Bugmann H, Groombridge B, et al. Mountain systems. In: Hassan R, Scholes R, Ash N, editors. Ecosystems and human well-being: current state and trends,. volume 1. Washington DC,: Island Press; 2005. p. 683–716. | 1 | Payne D, Spehn EM, Snethlage M, Fischer M. Opportunities for research on mountain biodiversity under global change. Curr Opin Environ Sustain [Internet]. 2017 Dec;29:40–7. Available from: doi: 10.1016/j.cosust.2017.11.001 | The same pattern is described in the most current paper (Payne et al 2017), which was also included in the first draft; so, Körner et al (2005) was retracted |
| 5 | Gradstein SR, Homeier J, Gansert D. The Tropical Mountain Forest Patterns and Processes in a Biodiversity Hotspot. Biodiversi. Gradstein SR, Homeier J, Gansert D, editors. Vol. Biodiversi, Tropical Mountain Forest: Patterns and Processes in a Biodiversity Hotspot. Göttingen: Göttingen Centre for Biodiversity and Ecology; 2008. 35–50 p. | 5 | Peters MK, Hemp A, Appelhans T, Becker JN, Behler C, Classen A, et al. Climate–land-use interactions shape tropical mountain biodiversity and ecosystem functions. Nature. 2019;568(7750):88–92 | A similar process is described in the most current paper (Peters et al 2019), which was also included in the first draft; therefore, Gradstein et al (2008) was retracted |
| 6 | Sarmiento FO. Anthropogenic change in the landscapes of highland Ecuador. Geogr Rev. 2002;92(2):213–34. | 4 | Young KR. Andean land use and biodiversity: Humanized landscapes in a time of change. Ann Missouri Bot Gard. 2009;96(3):492–507. | A similar finding was described in the most current paper (Young 2009), which was also included in the first draft; therefore, Sarmiento’s reference was retracted |
| 11 | Young KR. Ecology of land cover change in glaciated tropical mountains. Rev Peru Biol. 2014;21(3):259–70. | 4 | Young KR. Andean land use and biodiversity: Humanized landscapes in a time of change. Ann Missouri Bot Gard. 2009;96(3):492–507. | The article from Young (2014) was retracted because another article by the same author (Young 2009) contextualizes the idea at a regional scale and it was also included in the first draft; so, Young (2014) was retracted |
| 15 | Wilson SJ, Coomes OT, Dallaire CO. The ‘ecosystem service scarcity path’ to forest recovery: a local forest transition in the Ecuadorian Andes. Reg Environ Chang. 2019;19(8):2437–51 | 8  12  13 | Madrigal-Martínez S, Miralles i García JL. Land-change dynamics and ecosystem service trends across the central high-Andean Puna. Sci Rep. 2019;9(1):1–12.  Ross C, Fildes S, Millington AC.  Land-use and land-cover change in the páramo of South-Central Ecuador, 1979-2014. Land. 2017;6(3).  Tapia-Armijos MF, Homeier J, Espinosa CI, Leuschner C, De La Cruz M. Deforestation and forest fragmentation in south Ecuador since the 1970s - Losing a hotspot of biodiversity. PLoS One. 2015;10(9):1–18. | The three references :8,12,13 (in the revised version) best exemplify the idea that is intended to be put forward, so Wilson’s reference was retracted. These references were included in the first draft |
| 16  77 | Millennium Ecosystem Assessment. Ecosystems and Human Well Being [Internet]. Washington, DC.; 2005. Available from:  http://www.who.int/entity/globalchange/ecosystems/ecosys.pdf%5Cnhttp://chapter.ser.org/europe/files/2012/08/Harris.pdf  Millennium Ecosystem Assesment. Ecosystems and Human Well-being [Internet]. Vol. 5, Ecosystems. 2005. 1–155 p. Available from:  http://www.who.int/entity/globalchange/ecosystems/ecosys.pdf%5Cnhttp://www.loc.gov/catdir/toc/ecip0512/2005013229.html | 3 | Millennium Ecosystem Assessment (MEA). Synthesis. Ecosystems and Human Well Being. Washington, DC.; 2005. | This reference was repeated in the reference list of the first draft; therefore, the repeated one was retracted |
| 17 | Lambin EF, Turner BL, Geist HJ, Agbola SB, Angelsen A, Bruce JW, et al. The causes of land-use and land-cover change: Moving beyond the myths. Glob Environ Chang. 2001;11(4):261–9. | 14 | Lambin EF, Geist HJ, Lepers E. Dynamics of land-use and land-cover change in tropical regions. Annu Rev Environ Resour. 2003;28:205–41. | The same finding is stated in the most current article of the same author (Lambin, Geist and Lepers 2003); therefore, references 17 & 21 were retracted from the manuscript |
| 21 | Geist HJ, Lambin EF. Proximate causes and underlying driving forces of tropical deforestation. Bioscience. 2002;52(2):143–50. |  |  |  |
| 9,  24 | Madrigal-Martínez S, Miralles i García JL. Land-change dynamics and ecosystem service trends across the central high-Andean Puna. Sci Rep. 2019;9(1):1–12.  Madrigal-Martínez S, Miralles i García JL. Land-change dynamics and ecosystem service trends across the central high-Andean Puna. Sci Rep. 2019;9(1):1–12. | 8 | Madrigal-Martínez S, Miralles i García JL. Land-change dynamics and ecosystem service trends across the central high-Andean Puna. Sci Rep. 2019;9(1):1–12. | This reference was repeated in the reference list of the first draft, therefore the repeated one was retracted |
| 25 | Kristensen P. The DPSIR Framework. In: Workshop on a comprehensive / detailed assessment of the vulnerability of water resources to environmental change in Africa using river basin approach.Nairobi, Kenya: UNEP Headquarters; 2004. | 23 | OECD. OECD environmental indicators: development, measurement and use. 2003. | The DPSIR framework was originally stated in the OECD (2003), thus Kristensen's reference was replaced. In addition, the latest version of the manuscript has more detail on the DPSIR conceptual framework, which was based on some complementary references (25-32). |
| 26  30 | GAD PM. Plan de Ordenamiento y Desarrollo Cantonal Pedro Moncayo [Internet]. 2015. p. 138. Available from: <http://www.pedromoncayo.gob.ec/documentos/ord2015/PDOT.pdf>  Gobierno Autonomo Descentralizado Pedro Moncayo. Plan De Ordenamiento Y DesarrolloTuristico. 2015. | 34  37 | Gobierno Autonomo Descentralizado Pedro Moncayo. Plan De Ordenamiento Y Desarrollo Turistico. 2015.  Gobierno Autonomo Descentralizado Pedro Moncayo. Plan De Ordenamiento Y DesarrolloTuristico. 2015. | This reference was repeated in the reference list of both drafts. In the latest version, we deleted the repeated one, therefore the reference 37 was retracted from the latest version of the manuscript. |
| 39  40  42 | Guan D, Gao W, Watari K, Fukahori H. Land use change of Kitakyushu based on landscape ecology and Markov model. J Geogr Sci. 2008;18(4):455–68.  Zhang B, Li S, Jia X, Gao L, Peng M. Adaptive Markov random field approach for classification of hyperspectral imagery. IEEE Geosci Remote Sens Lett. 2011;8(5):973–7.  Cheng L, Xia N, Jiang P, Zhong L, Pian Y, Duan Y, et al. Analysis of farmland fragmentation in China Modernization Demonstration Zone since “ Reform and Openness”: A case study of South Jiangsu Province. Sci Rep [Internet]. 2015;5(May):1–11. Available from:  http://dx.doi.org/10.1038/srep11797 | 48  49 | Liping C, Yujun S, Saeed S. Monitoring and predicting land use and land cover changes using remote sensing and GIS techniques - A case study of a hilly area, Jiangle, China. PLoS One. 2018;13(7):1–23  Hamad R, Balzter H, Kolo K. Predicting land use/land cover changes using a CA-Markov model under two different scenarios. Sustain. 2018;10(10):1–23. | These references were replaced by more up-to-date citations which better exemplify the applicability of transition probabilities from Markov models in land use change analysis |
| 47 | Aik DHJ, Ismail MH, Muharam FM, Alias MA. Evaluating the impacts of land use/land cover changes across topography against land surface temperature in Cameron Highlands. PLoS One [Internet]. 2021;16(5 May):1–26. Available from: http://dx.doi.org/10.1371/journal.pone.0252111 | 42 | Jin DH, Ismail MH, Muharam FM, Alias MA. Evaluating the impacts of land use/land cover changes across topography against land surface temperature in Cameron Highlands. PLoS One [Internet]. 2021;16(5 May):1–26. Available from: doi: 10.1371/journal.pone.0252111 | This reference contained an error in the surname of the first author in our bibliographic database; then, the error was corrected and this citation appears with the correct surname (Jin) in the revised manuscript |
| 54    55 | Karger DN, Conrad O, Böhner J, Kawohl T, Kreft H, Soria-Auza RW, et al. Climatologies at high resolution for the Earth land surface areas. Sci Data. 2017;4 170122.  D.N. K, Conrad O, Böhner J, Kawohl T, Kreft H, Soria-Auza RW, et al. Data from: Climatologies at high resolution for the earth’s land surface areas | 59 | Karger DN, Conrad O, Böhner J, Kawohl T, Kreft H, Soria-Auza RW, et al. Climatologies at high resolution for the Earth land surface areas. Sci Data. 2017;4 170122. | This reference was repeated in the reference list of the first draft because there was an error in our bibliographic database |
| 61 | Halliday A, Glaser M. Management Perspective On Social Ecological Systems. Res Hum Ecol.694 2011;18(1):1–18. | 8 64 | Madrigal-Martínez S, Miralles i García JL. Land-change dynamics and ecosystem service trends across the central high-Andean Puna. Sci Rep. 2019;9(1):1–12.  Deler J-P, Gomez N, Portais M. El manejo del espacio en Ecuador. In: Geografía básica del Ecuador Tomo I Geografía Histórica [Internet]. Quito: Centro Ecuatoriano De Investigación Geográfica; 1983. p. 267. Available from: http://horizon.documentation.ird.fr/exl-doc/pleins_textes/divers11-03/03421.pdf | This reference was removed in editing; therefore, it was retracted |
| 64 | De S, Andes L, Del U, Charles E, Crissman C. La Agricultura En Los P Aramos: Estrategias Para El [Internet]. Lima; 2003. Available from:  http://condesan.org/mtnforum/sites/default/files/publication/files/cds-  01_agricultra_en_paramos_opt.pdf | 66  67 | Camacho M. Los páramos ecuatorianos: Caracterización y consideraciones para su conservación y aprovechamiento sostenible. An la Univ Cent del Ecuador 372. 2013;76–92.  Garavito L. Los páramos en Colombia, un ecosistema en riesgo. Ingeniare, Univ Libr [Internet]. 2015;(19):127–36. Available from: http://www.unilibrebaq.edu.co/ojsinvestigacion/index.php/ingeniare/article/view/704 | This reference was retracted because its findings were described in detail in the two references (Camacho 2013 and Garavito 2015), which were already included in the first version of the manuscript |
| 74 | Westphal C, Bommarco R, Carré G, Lamborn E, Morison M, Petanidou T, et al. Measuring bee diversity in different European habitats and biogeographic regions. Ecol Monogr. 2008;78(4):653–71. |  |  | This reference was removed in editing; therefore, it was retracted |
| 91 | Franco A, Sobral B, Silva A, Wall D. Amazonian deforestation and soil biodiversity. Conserv Biol. 2018;0(0):1–11. |  |  | This reference was removed in editing; therefore, it was retracted |

In addition, we did a final proofreading of the entire manuscript and

found some spelling errors and typos that were corrected in this version; we also made some final edits in the text to clarify some ideas. These edits are explained below:

| **Lines** | **Corrections** |
| --- | --- |
| 29-30 | We replaced holistic framework by Driver-Pressure-State-Impact-Response (DPSIR) framework |
| 43 | The word ‘as’ was removed |
| 51-52 | ‘The so called’ was replaced by ‘through the provision of’. Also, we capitalized Ecosystem Services to use its abbreviation throughout the manuscript. To improve the connection of ideas, we moved the sentence that previously occupied lines 52 and 53 to line 51, and by making this change we modified the order in which the references were entered. |
| 59 | The word ‘however’ was added |
| 61 | The word ‘however’ was deleted |
| 72-73 | We eliminated the marks (underlines) left by the track changes made by one of the reviewers in the initials of Land Use Land Cover. |
| 127 | The closing of a parenthesis erroneously included was eliminated. |
| 129 | A comma was replaced by the conjunction ‘y’.. |
| 159 | We rephrased the last sentence as follows: ‘where these aspects have been integrated into the DPSIR approach for this study’ |
| 213 | There was a typo in the first word of this line (publically), it was corrected (publicly) |
| 226 | The preposition OF was missing in the sentence and also the official data source was incomplete as it was generated by two institutions MAE and MAGAP. Therefore, MAE was added. |
| 269 | The closing of a parenthesis erroneously included was eliminated. |
| 327 | We added the preposition ‘and’ before pastures, because it was missing in the sentence. |
| 400, 497 | We removed the highlights of references (S1 Fig) and (S2 Fig) which were marked by one of the reviewers. We decided to keep all figures in the supplemental material. |
| 430 | We deleted OF ANALYSIS, since it was redundant in the sentence. |
| 431 | To better phrasing, we replaced OF ANALYSIS, by STUDIED. |
| 461 | There was a mistake in the referred model, therefore we replaced EXPANSION by LOSS. |
| 466 | We eliminated the marks (a lateral line) left by the track changes made in the previous version when the Table 3 was inserted. |
| 510-511 | The words ‘at present’ were deleted in line 510 and the following text was added at the end of line 511 ‘during the last several decades’ |
| 513-514 | We replaced ‘studied area depicts’ by the study area of Pedro Moncayo represents’ |
| 516 | The words ‘which is’ were deleted |
| 523 | The word ‘in’ was deleted after the reference 12 |
| 525 | The words ‘respectively’ were added |
| 537 | A semicolon (;) was replaced by a period (.) before the word this |
| 547 | We replaced ‘great’ by ‘relatively high’ |
| 584 | We replaced ‘better environment by ‘subsidy’ |
| 579 | The word FACILITIES was repeated in the same sentence; therefore, we deleted the first one and replace it by the word AIRPORT in plural. |
| 628 | To better phrasing, we replaced STUDIES, by FINDINGS. |
| 636-637 | At the end of the paragraph we added ‘representing an increasing pressure on natural resources to sustain livelihoods in the region’ |
| 653 | We replaced ‘other studies have suggested’ by ‘for example’ |
| 654 | We added ‘have been cited’ |
| 655 | We replaced ‘but also due to diffusion of’ by ‘increased access to’ |
| 651 | We removed the hyphen after the word ‘land’ and before the word ‘use’ to keep the same term consistent throughout the manuscript. |
| 711 | We replaced the word ‘is by ‘was’ |
| 722-724 | We included a text in the acknowledgements section to thank the local authorities for providing geo-referenced information of the studied region. |

Finally, we made some adjustments in the format of the reference list. For all the references that included Institutions as authors of the information, we first included the full name of the institution, followed by their respective abbreviation in parenthesis. The following references in the Bibliography contain these format adjustments: References: 3, 23, 37, 38, 45, 50,57 and 85.

We hope that with these minor corrections we will have fully complied with the requirements of PLOS ONE to publish our manuscript in this renowned scientific journal.

Sincerely,

Paulina Guarderas, M. Sc.

Corresponding Author

| Facultad de Ciencias Biológicas,  Universidad Central del Ecuador  Numa Pompilio Liona S/N y Yaguachi,  QUITO, Ecuador  E-mail: apguarderas@uce.edu.ec  Cel: 593 992403093 | Additional Contact:  Biodiversity and Landscape Unit,  Gembloux Agro Bio-Tech,  University of Liège  Passage des Dport s 2, 5030  GEMBLOUX, Belgique  E-mail: ap.guarderas@doct.uliege.be |
| --- | --- |
